# A Fluorogenic Far Red-Emitting Molecular Viscometer for Ascertaining Lysosomal Stress in Live Cells and *Caenorhabditis elegans*

**DOI:** 10.3389/fchem.2022.840297

**Published:** 2022-03-11

**Authors:** Akshay Silswal, Ashutosh Kanojiya, Apurba Lal Koner

**Affiliations:** Bionanotechnology Lab, Department of Chemistry, Indian Institute of Science Education and Research Bhopal, Bhopal, India

**Keywords:** far red-emitting fluoroprobe, molecular rotor, cellular viscometry, lysosomal probe, cancer

## Abstract

The cellular physiochemical properties such as polarity, viscosity, and pH play a critical role in cellular homeostasis. The dynamic change of lysosomal viscosity in live cells associated with different environmental stress remains enigmatic and needs to be explored. We have developed a new class of Julolidine-based molecular viscometers with an extended *π-*conjugation to probe the lysosomal viscosity in live cells. High biocompatibility, pH tolerance, and the fluorogenic response with far red-emission (>600 nm) properties make these molecular viscometers suitable for live-cell fluorescence imaging in *Caenorhabditis elegans*. Among these probes, **JIND-Mor** is specifically designed to target lysosomes *via* simple modification. The real-time monitoring of lysosomal viscosity change under cellular stress was achieved. We believe that such a class of molecule viscometers has the potential to monitor lysosomal health in pathogenic conditions.

## Introduction

The microenvironmental cellular properties play an important role in biological function (Chambers et al., 2018). The unusual changes of polarity, viscosity, potential difference, and membrane tension are associated with many disorders and diseases (Yang et al., 2014; Pal et al., 2020; Biswas et al., 2021; Pal et al., 2021). The anomalous changes in cellular fluidity are considered as one of the vital indicators of neurodegenerative disorders, atherosclerosis, diabetes, and even cancer (Junttila and de Sauvage, 2013). In such diseases, the subcellular viscosity changes significantly, as several important biochemical processes depend on it (Gao et al., 2019; Ma et al., 2020). Therefore, monitoring organelle viscosity is immensely important for disease diagnosis (Klymchenko, 2017).

The lysosome, a membrane-bound spherical organelle, is known as the digestive compartment of the cells and plays an important role in cellular homeostasis (Lawrence and Zoncu, 2019). It is an acidic compartment and contains approximately 60 hydrolytic enzymes for breaking all sorts of biomolecules (Lawrence and Zoncu, 2019). The viscosity of lysosome and lysosome-related organelles (LROs) is closely associated with overall animal health and a key indicator of its functionality (Li et al., 2018; Tan et al., 2019; Cai et al., 2021). Therefore, sensitive monitoring of their viscosity with a specific nano-sized molecular viscometer is essential for understanding cellular health.

Among the existing approaches for cellular viscosity determination, intramolecular charge transfer (ICT) dye-based molecular rotors are mostly preferred (Haidekker and Theodorakis, 2016; Ye et al., 2021). The excited CT state can be rapidly deactivated through intramolecular rotation about the donor–acceptor bond (Su et al., 2017). However, the restricted motion due to the high viscosity of the surrounding medium results in a fluorogenic response (Su et al., 2017). An organelle selective and background-free molecular rotor with a fluorogenic response is mostly preferred owing to their rapid noninvasive measurement and spatiotemporal monitoring (Kuimova et al., 2008; Wang et al., 2013). Julolidine-based molecular rotor such as 9-(dicyanovinyl)-julolidine, 9-(2-carboxy-2-cyanovinyl)julolidine, and their suitable derivatives are well explored for quantifying the cellular viscosity (Kung and Reed, 1989; Haidekker et al., 2001; Shao et al., 2011). However, they suffer from small Stokes shift and high-energy excitation, which limits their applicability for *in vivo* applications (Haidekker et al., 2001).

**SCHEME 1:**
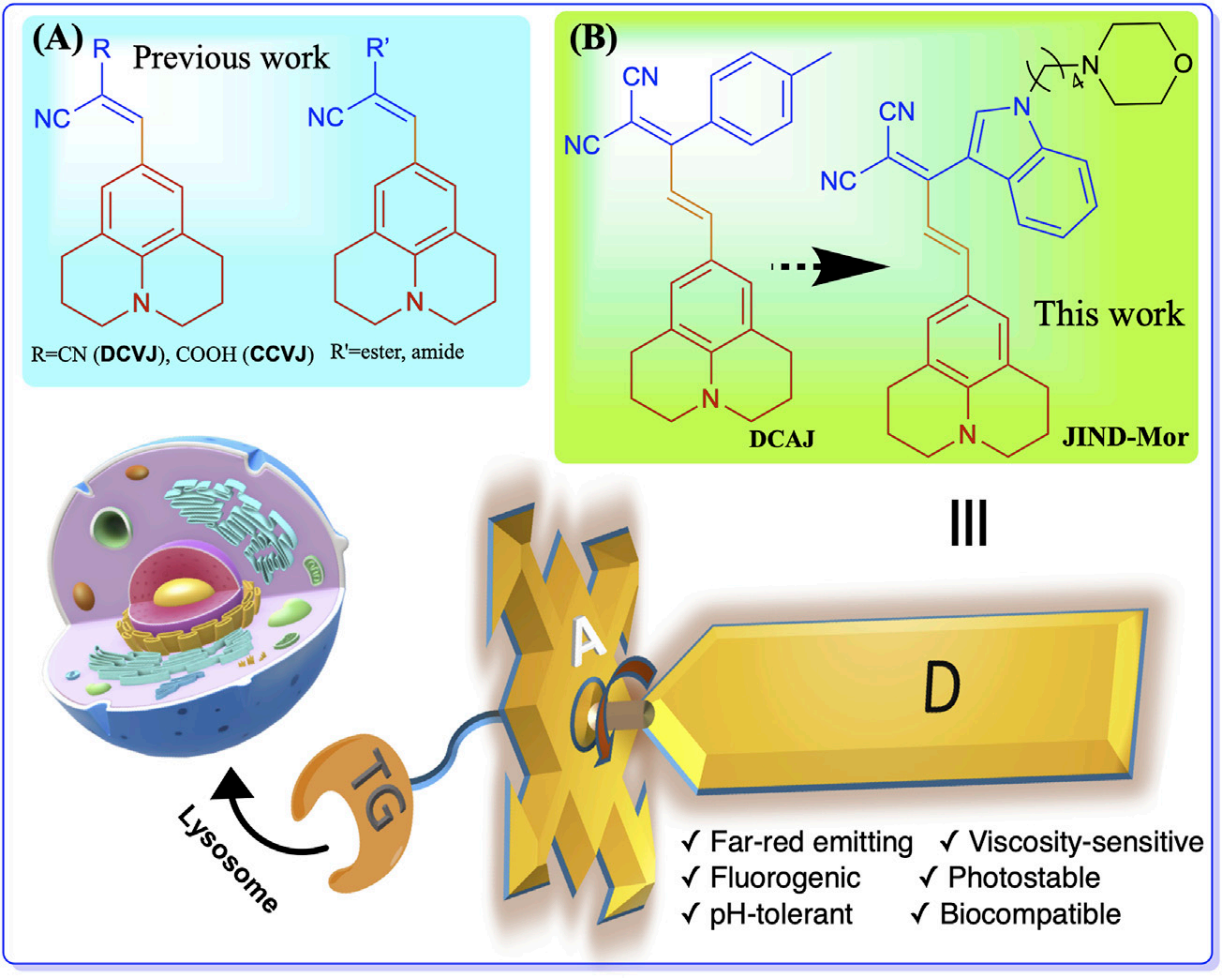
Structure of molecular rotors **(A)** previously developed julolidine-based, **(B) DCAJ**, and **JIND-Mor** in this work. Molecular rotor has three components-donor **(D)** group, a bulky acceptor **(A)** unit, and a lysosome selective targeting group remotely connected to acceptor.

To overcome these limitations, we have developed a far-red emitting pH-tolerant molecular viscometer **DCAJ** with a fluorogenic response. The synthesized molecules show a large Stokes shift of approximately 150 nm in water. Furthermore, to obtain a more sensitive molecular-rotor (**JIND**), we have introduced a bulkier indole group by replacing the tolyl group. Later, the indole moiety is suitably functionalized with a well-explored lysosome targeting group morpholine (**JIND-Mor**) Scheme 1. Morpholine-appended fluorescent probes are known to localize selectively inside lysosomal compartment due to its protophilic nature associated with the low p*K*_a_ value in the range of 5–6 (Wang et al., 2013; Li et al., 2018; Kong et al., 2019; Biswas et al., 2021).

## Materials and Methods

### Synthesis and Photophysical Measurements

All reagents and solvents were purchased from commercial sources and used without further purification. Thin-layer chromatography was performed using Merck Silica gel 60 F-254 pre-coated plates and visualized using a thin-layer chromatographic chamber equipped with ultraviolet (UV) (*λ* = 254/365 nm) and visible light. Silica gel from Merck (particle size 100–200 mesh) and neutral alumina from Rankem were used for column chromatography. ^1^H and ^13^C nuclear magnetic resonance spectra were recorded on Bruker 400- and 500-MHz spectrometers. High-resolution mass spectrometry data were recorded on MicrOTOF-Q-II mass spectrometer using acetonitrile as the solvent. All absorption spectra and fluorescence measurements were carried out using SHIMADZU UV-1800 spectrophotometer and HORIBA JobinYvon fluorimeter (fluorolog-3) using 1-cm path length quartz cuvettes.

The viscosity of different weight percentages of water/glycerol mixture was calculated from a previous report by Cheng (2008). The different weight percentage of water/glycerol mixture was prepared 10 ml each from this solution, 2 ml taken, and dye was added and mixed well using vortex (SCILOGEX vortex mixture MX-S) for 5 min, then immediately spectra were recorded at a fixed temperature of 25°C.

### Cell Culture and Imaging

Dulbecco’s modified Eagle medium (DMEM), trypsin, antibiotic cocktail, and fetal bovine serum (FBS) were purchased from HiMedia (USA). Lyso-Tracker Green and MitoTracker Green were purchased from Thermo Fisher Scientific (United States). Our laboratory synthesized ER Tracker Green previously (Dutta et al., 2020). The 35-mm glass bottom imaging dishes were obtained from Ibidi (Germany, Cat# S28 81158). All the confocal microscopy imaging was performed with an Olympus FV3000 confocal laser scanning microscope. BHK-21 and U-87 MG cells were obtained from the National Centre for Cell Science, Pune, India, and were grown in a 25-cm^2^ cell culture flask (Corning, United States) using DMEM (phenol red-free) containing 10% (v/v) FBS and 1% (v/v) antibiotic cocktail in 5% CO_2_ at 37°C in a CO_2_ incubator. For imaging purposes, cells were grown to 75–80% confluency in the 35-mm glass bottom imaging dishes (170 ± 5 *µ*m) in DMEM with 10% FBS. The cells were washed twice with phosphate-buffered saline (PBS; pH 7.4) containing 5-mM MgCl_2_. For the colocalization experiment, the cells were co-incubated with 0.2 µM of the **JIND-Mor**, and 300 nM of LysoTracker Green, 300-nM MitoTracker Green, and 2.5-*µ*M ER Tracker Green for 15 min, and washed with PBS (pH 7.4) containing 5-mM MgCl_2_ twice before imaging. For viscosity tracing, firstly, U-87 MG cells were incubated with 0.2-*µ*M **JIND-Mor** for 15 min and washed twice with PBS (pH 7.4) containing 5-mM MgCl_2_, then 50-*µ*M dexamethasone (Dexa) was added and immediately observed on the confocal microscope for 60 min. Quantification of the lysosomal and *Caenorhabditis elegans* intensity was done using Image J software.

### *Caenorhabditis elegans* Culture

Hermaphrodite worms were grown in a nematode growth medium (NGM) at 20°C. For staining, *C. elegans* were synchronized and grown to young adult stage in NGM treated with 10-*µ*M **JIND-Mor** for 60 h in 20°C. To induce osmotic stress, worms were synchronized and grown at 20°C until the first day of adulthood. The animals were transferred to NGM plates containing 200-mM NaCl for 8 h. Here, NGM plates containing 50-mM NaCl were used as control conditions. They were transferred to an agar pad on a glass slide and paralyzed using 5-mM levamisole and imaged under a confocal microscope. Quantification was done using Image J software using three different worms’ images.

### DFT Calculation

The theoretical calculations were performed using the Gaussian 09 suite of the quantum chemical program (Frisch et al., 2009). Ground-state geometry optimization was performed with Becke’s three-parameter hybrid exchange functional with Lee–Yang–Parr correlation (B3LYP functional) using 6-311G as a basis set.

## Results and Discussion

### Synthesis of Molecular Rotors

**SCHEME 2:**
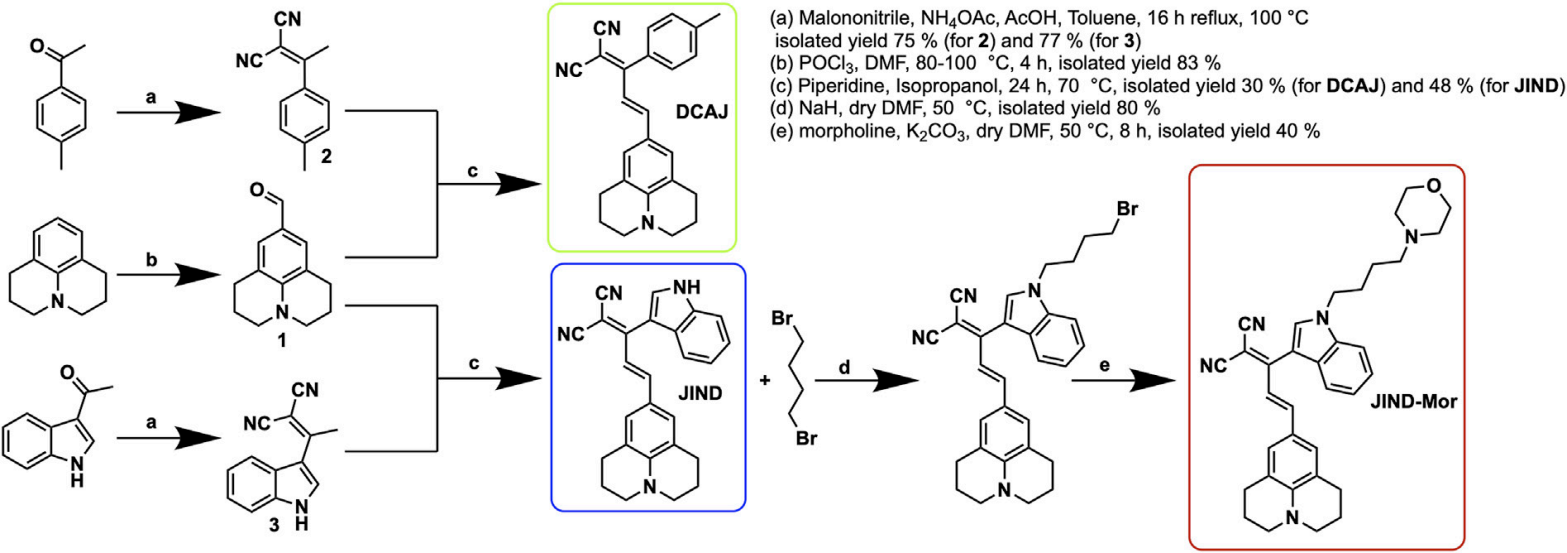
Synthesis route for compound **DCAJ**, **JIND**, and **JIND-Mor**

**DCAJ** was synthesized by condensation of compounds **1** and **2** (Scheme 2; see SI for details). Compound **1** was synthesized from the formylation of Julolidine using Vilsmeier–Haack reaction in an 83% isolated yield. However, compound **2** was obtained from the reaction of 4-methylacetophenone and malononitrile in a 75% yield. The reaction of 3-acetylindole and malononitrile yielded compound **3**. Now to get **JIND**, compounds **1** and **3** were reacted in the presence of piperidine in isopropanol. **JIND** was further reacted with 1,4-dibromobutane to get the compound **5**, and it was subsequently reacted with morpholine in dry dimethylformamide to obtain **JIND-Mor** in a 40% isolated yield. All compounds were characterized with nuclear magnetic resonance spectroscopy and mass spectrometry (see SI, Supplementary Figures S1–S17).

### Solvent Polarity-Dependent Optical Properties

**FIGURE 1:**
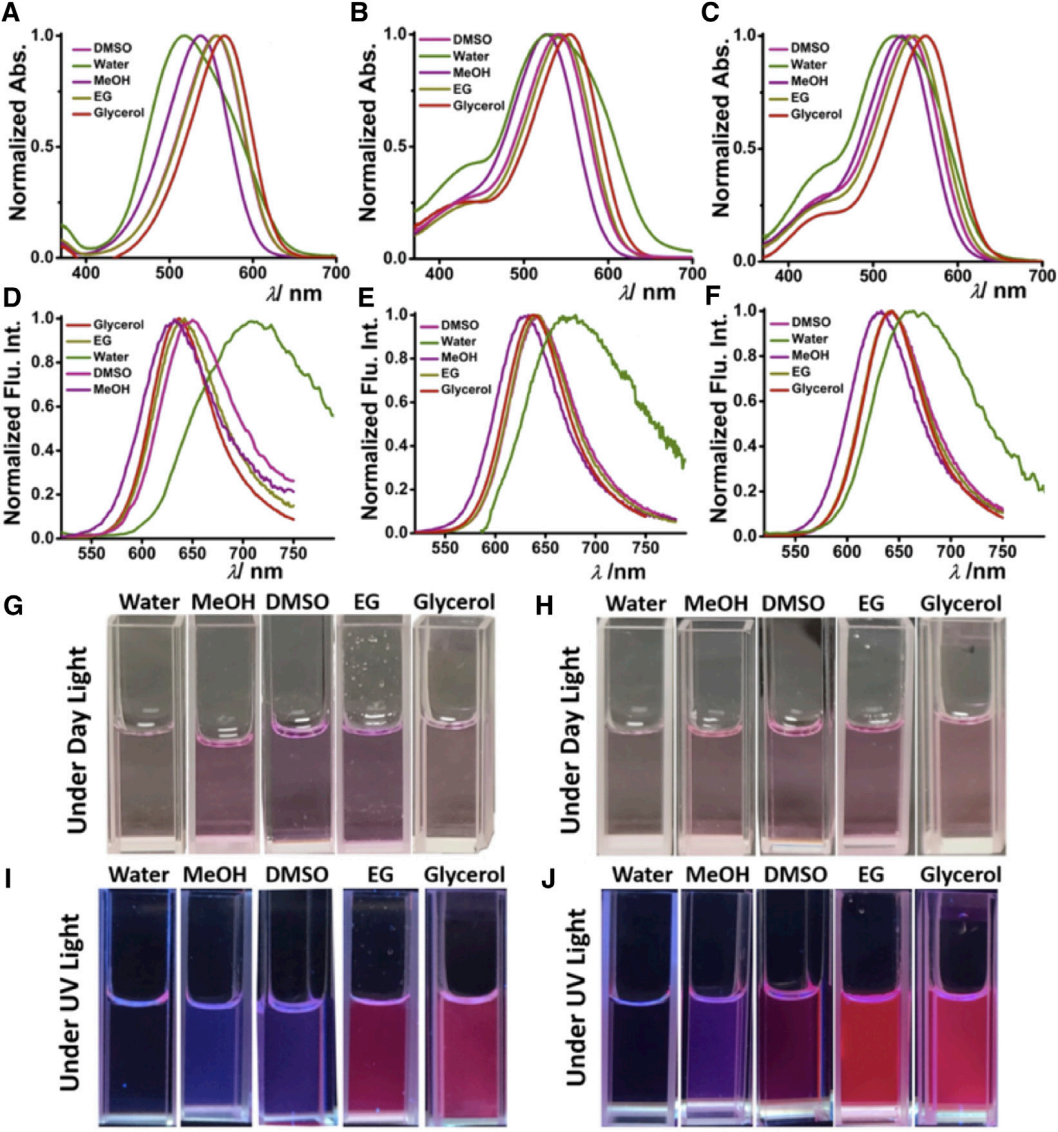
Solvent-dependent ultraviolet–visible. Absorption [**(A) DCAJ**, **(B) JIND**, and **(C) JIND-Mor**] and fluorescence properties [**(D) DCAJ**, **(E) JIND**, and **(F) JIND-Mor**] of 5-µM dye. Cuvette images of **DCAJ** (bottom left) **(G)** under daylight, **(I)** under ultraviolet light (∼365 nm), and **JIND** (bottom right) **(H)** under daylight **(J)** under UV light (∼365 nm).

The solvent-dependent optical properties, such as ultraviolet–visible, and absorption and fluorescence of **DCAJ**, **JIND**, and **JIND-Mor** were investigated in detail (Supplementary Table S1; Figures 1A–F, Supplementary Figures S18–S20). A red-shift in the absorption and emission maxima of these compounds with solvent polarity confirms their ICT property. The ICT character of these molecules is also evident from the fragment molecular orbital calculation, as electron density in highest occupied molecular orbital is mostly distributed on julolidine (donor) and on malononitrile (acceptor) in the case of lowest unoccupied molecular orbital (Supplementary Figure S21). In water, these molecules have an absorption maximum of around 530 nm with an emission maximum of around 680 nm (Figure 1B). As shown in Figures 1G–J, depending on the solvent polarity, the color of the solution of **DCAJ** and **JIND-Mor** changes under visible and UV (365 nm) light. The probes are highly photostable, thermostable, and pH tolerant, as assessed from their unaltered fluorescence intensity (see SI, Supplementary Figure S22–S24).

### Viscosity-Dependent Optical Properties

**FIGURE 2:**
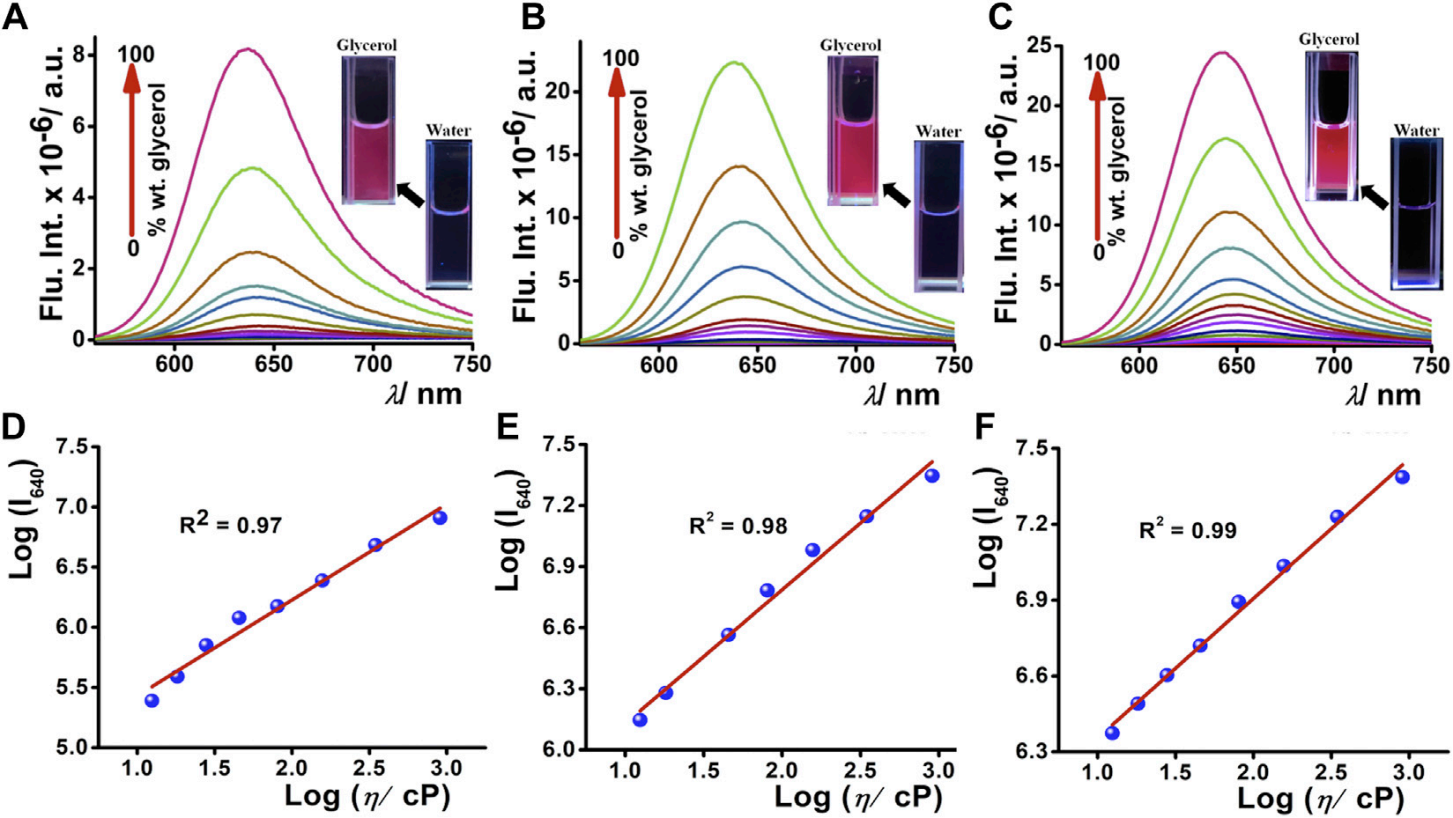
Fluorogenic response of 5-µM **(A) DCAJ**, **(B) JIND**, and **(C) JIND-Mor** with increasing concentration of glycerol percentage in water; inset shows fluorescence images of 5 µM compound in water and in glycerol under 365-nm light exposure. Double logarithmic plot of fluorescence intensity of solution and its viscosity (in centipoise) **(D) DCAJ**, **(E) JIND**, and **(F) JIND-Mor** shows linear dependence.

**FIGURE 3:**
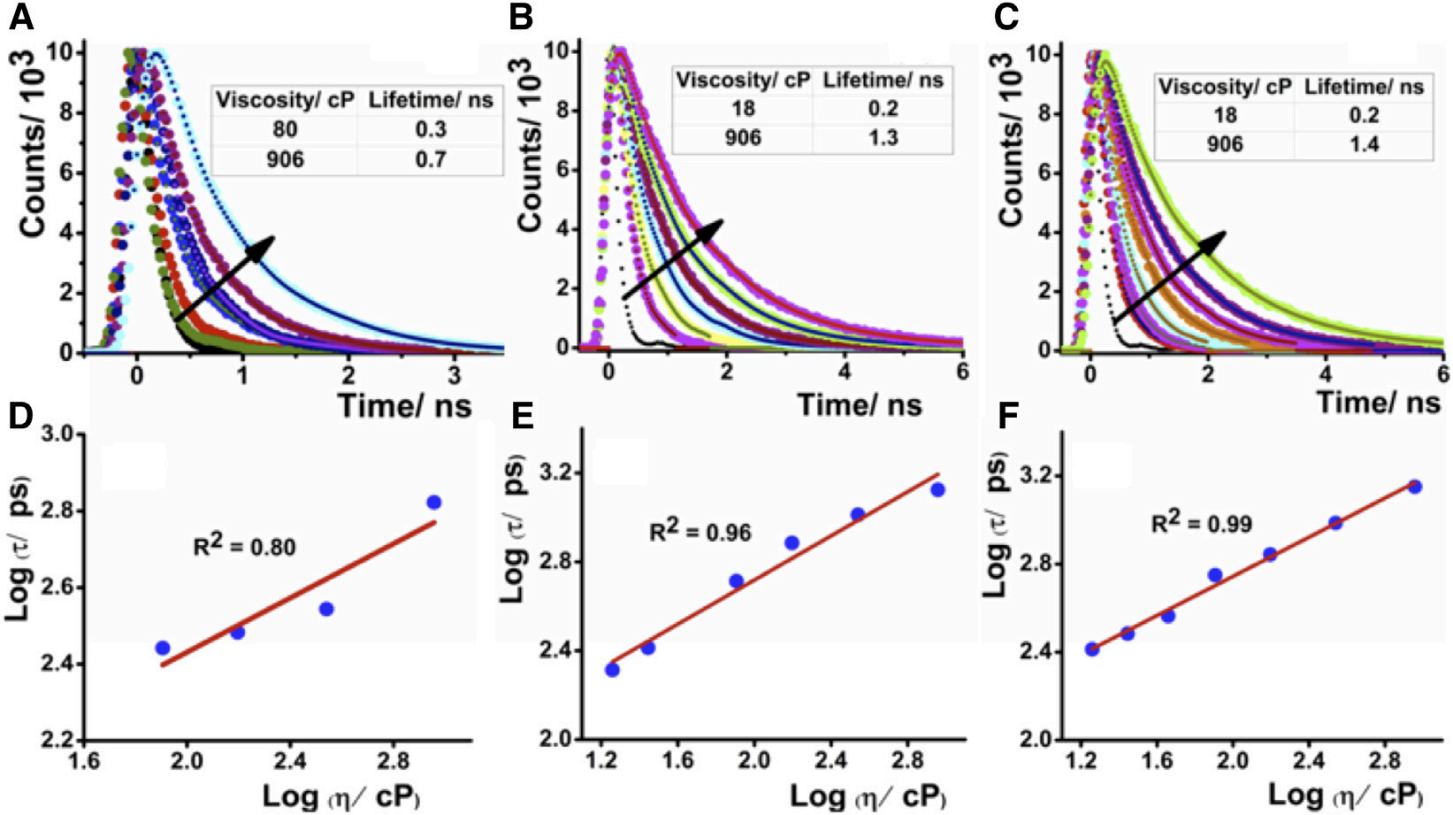
Time-resolved fluorescence decay of **(A) DCAJ**, **(B) JIND**, and **(C) JIND-Mor** in water–glycerol mixture with increase of solution viscosity. Linear dependence of logarithmic value of lifetime in picosecond of compounds vs logarithmic value of solution viscosity for **DCAJ (D)**, **JIND (E)**, and **JIND-Mor (F)**. Values are fitted with equation of a straight line.

To apprehend the molecular rotor properties of **DCAJ**, **JIND**, and **JIND-Mor**, we have investigated the viscosity-dependent change in fluorescence intensity and lifetime. In highly viscous solvents such as ethylene glycol and glycerol, we observed a noteworthy >150 times intensity enhancement, without any correlation with solvent polarity (Supplementary Figure S25). Such enhancement in the fluorescence intensity prompted us to investigate the viscosity-dependent emission properties of these compounds in detail. We observed a fluorogenic response upon moving from pure water (viscosity = 0.89 cP) to pure glycerol solution (viscosity = 905 cP), as shown in Figures 2A–C. A distinct visible color change from a nearly nonfluorescent state to a highly fluorescent state is also observed (inset of Figures 2A–C), signifying the molecular rotor nature of the compounds. We have used the Forster–Hoffmann equation to quantify the relation between fluorescence quantum yield and the viscosity of the solution (Wu et al., 2013), *i.e.*, $\phi_{f}=C\eta^{x},$ taking logarithm on both sides, we can get: $\log\left(\phi_{f}\right)=\log C+x\log\eta$ and for a lifetime $\log\left(\tau_{f}\right)=\log C^{'}+x\log\eta$ , where $\phi_{f}$ is the fluorescence quantum yield, C and C' are the constants, $C^{'}=C\times \left(k_{r}^{-1}\right)$ , $k_{r}=$ radiative rate constant, and η = the viscosity of the medium. The restriction of molecular motion on moving from low to high viscous environment suppresses the non-radiative pathways and results in fluorescence enhancement. The double logarithmic plot of log (Intensity) measured at emission maxima with log (viscosity) of all these compounds well fitted with the equation discussed earlier, as shown in Figures 2D–F and Supplementary Figure S26. The fluorescence lifetime of these molecular rotors is also enhanced by increasing the medium viscosity. The fluorescence lifetime of these molecules in water is quite short and increases gradually upon increasing the viscosity of the medium (Figures 3A–C). The lifetime of **DCAJ** in glycerol is the shortest among all these rotors. On moving from **DCAJ** to **JIND**, the fluorescence lifetime increases upon substitution with the bulkier group, as evident from their lifetime in glycerol. The lifetime increases linearly with the viscosity of the medium (Figures 3D–F) as envisioned. The increased stiffness of the slope from this linear dependence for **DCAJ** to **JIND-Mor** confirms their improved sensitivity upon increases in bulkiness.

### Live-Cell Imaging and Subcellular Viscosity Measurement

**FIGURE 4:**
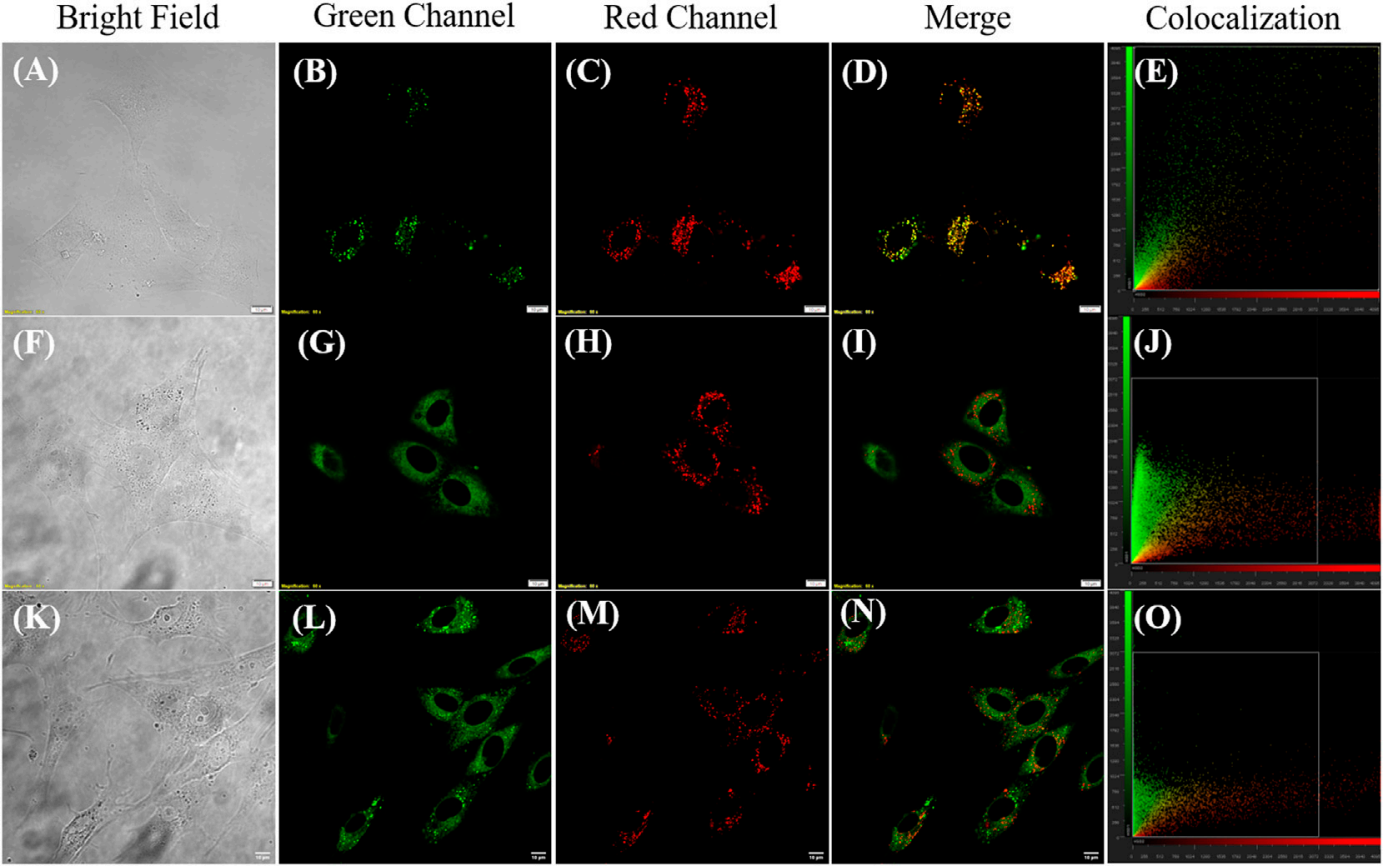
Confocal laser scanning microscope images of BHK-21 cells co-stained with 0.2–µM **JIND-Mor**, 0.3-µM LysoTracker Green, 0.3-µM MitoTracker Green, and 2.5-µM ER Tracker (**NBD-Bu**) (Dutta et al., 2020) for 15 min **(A**,**F,K)** Bright field; Green Channel (Ex: 488 nm, Em: 500–530 nm) **(B)** LysoTracker Green, **(G)** MitoTracker Green, and **(L)** ER Tracker (**NBD-Bu**); Red Channel (Ex: 561 nm, Em: 570–670 nm) **(C,H**,**M) JIND-Mor**; Merge image **(D)** of **(B,C)**, **(I)** of **(G,H)**, **(N)** of **(L,M)**; Scatter plot showing Pearson’s correlation coefficient of **(E)** 0.87 ± 0.02 with LysoTracker Green, **(J)** 0.32 ± 0.05 with MitoTracker Green, and **(O)** 0.20 ± 0.04 with ER Tracker (scale bar = 10 µm).

Furthermore, **JIND-Mor** was selected as a potential molecular rotor for monitoring viscosity changes in a living system. To start with, we have investigated the cellular toxicity of **JIND-Mor** in noncancerous cells (BHK-21) using a 3-(4,5-dimethylthiazol-2-yl)-2,5-diphenyl-2H-tetrazolium bromide assay. The IC_50_ value is more than 5 µM, and more than 70% of cells were viable even after 24 h (Supplementary Figure S27). After that, we have investigated the localization of **JIND-Mor** in the cellular compartments *via* live-cell fluorescence imaging using confocal laser scanning microscopy. To our pleasure, **JIND-Mor** selectively localizes in the lysosomal compartments of cells. The selectivity was assessed using a commercially available lysotracker green dye, known to localize selectively in the lysosome (Figures 4A–E, top panel, Supplementary Figure S28). The high Pearson-correlation coefficient of 0.87 establishes the selectivity. Furthermore, we investigated the localization with other organelle trackers such as mitochondrion and ER (middle and bottom panel of Figure 4). Contrarily, the Pearson coefficients obtained for ER (0.20) and mitochondrion (0.32) were quite low (Figures 4J,O). A comparison of the previously reported molecular rotor for lysosomal viscosity determination is provided in Supplementary Table S2.

### Stress-Induced Viscosity Measurement of Lysosome and *Caenorhabditis elegans*

**FIGURE 5:**
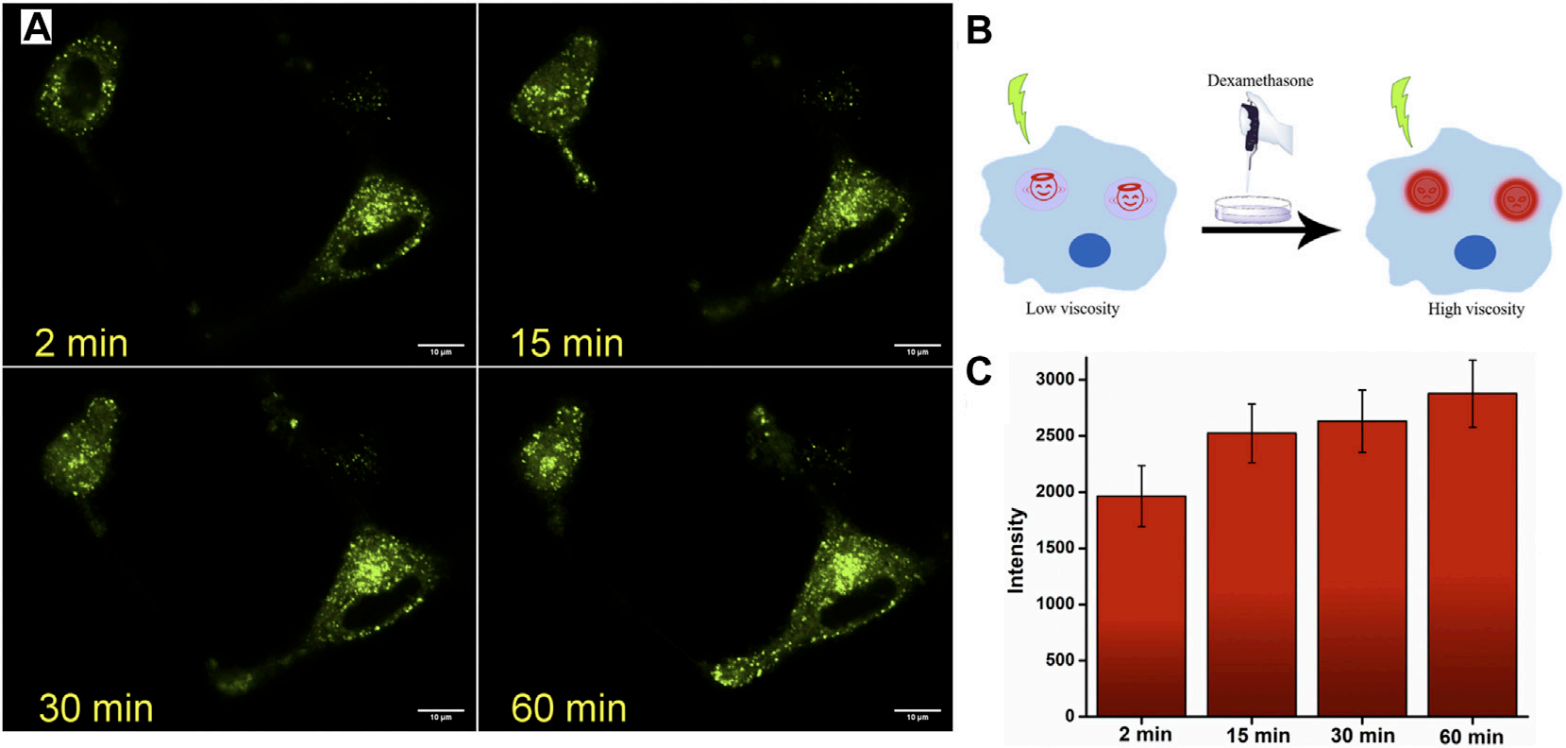
**(A)** Confocal laser scanning microscope images of U-87 MG cell lines stained with 0.2-µM **JIND-Mor** and then stimulated with 50-µM dexamethasone for representative period. **(B)** Pictorial representation of enhancement of fluorescence on restricting rotation of molecules by increasing viscosity through stimuli dexamethasone. **(C)** Quantification of dexamethasone induced an increase in fluorescence intensity (scale bar = 10 µm).

After establishing the lysosome-specific localization of **JIND-Mor**, we monitored the lysosomal viscosity change in glioblastoma (GBM), a known fast-growing and aggressive cancer cell (Taylor et al., 2019). It is established that cellular viscosity has strong influences on their progression, invasion, and morphological stability (Streitberger et al., 2020). Therefore, the determination of the lysosomal viscosity of GBM can provide useful information for its diagnosis and treatment (Perini et al., 2020). To assess the temporal changes of lysosomal viscosity in human GBM cells (U87-MG) using **JIND-Mor**, we have used Dexa as the stimulation reagent. Dexa acts as a lysosomal membrane stabilizer and an inhibitor of lysosomal enzymatic release, which causes an increase in the lysosomal viscosity (Yang et al., 2020). Therefore, the dynamic fluorescence change of the lysosomal compartment of U87-MG cells was measured after treating with **JIND-Mor** for 15 min and then stimulated with 50 µM of Dexa (Figure 5A). The fluorescence enhancement clearly indicated an increase in lysosomal viscosity upon treatment with Dexa without interrupting the lysosomal structural integrity (see Figure 5B). Furthermore, we have quantified the time-dependent fluorescence intensity of lysosomes (Figure 5C). The results discussed earlier indicate that **JIND-Mor** is potent for selective lysosomal localization and determination of viscosity change in live conditions.

**FIGURE 6:**
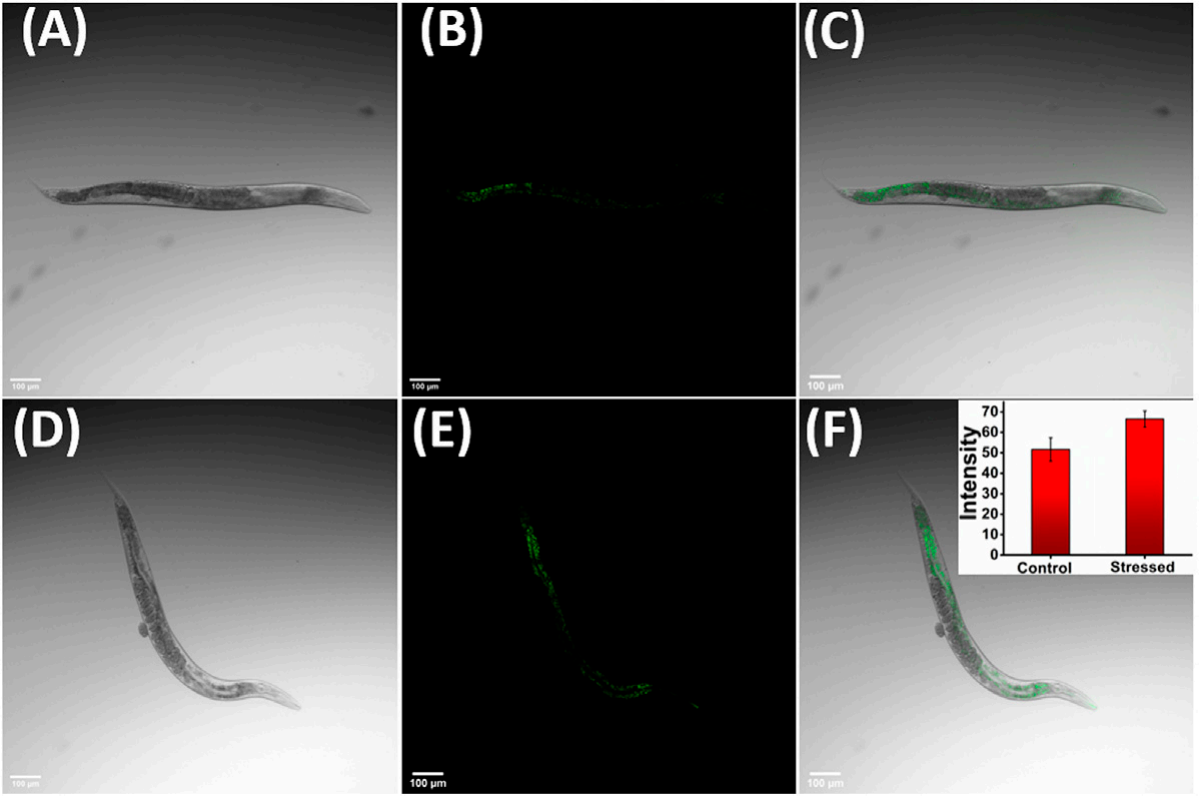
Osmotic stress-induced viscosity changes in *C. elegans*. Left: Bright field. Middle: Fluorescent images. Right: Merged image showing *in vivo* distribution of **JIND-Mor**. *C. elegans* incubated with 10-µM **JIND-Mor** under healthy conditions **(A–C)** and stressed conditions **(D–F)** (scale bar = 100 *µ*m). Inset: Quantification of stress-induced fluorescence increase (*n* = 3, error bar showing standard deviation).

To further validate the applicability of **JIND-Mor** for imaging lysosome-related organelle stress in *C. elegans*, they were grown to young adult stage in nematode growth medium with 10-µM **JIND-Mor** for 60 h at 20°C. The brighter green fluorescence under osmotic stress-induced conditions (Figures 6A–F) clearly indicates the increment in gut granules viscosity. These results revealed the potential applicability of **JIND-Mor** for viscosity sensing in living *C. elegans*.

## Conclusion

In summary, we have developed Julolidine-based far-red emitting molecular rotors with large Stokes-shift for probing lysosomal viscosity. **JIND-Mor** is highly photostable, pH-tolerant, and biocompatible for selective lysosomal localization and monitoring lysosomal stress in live cells and in *C. elegans*. We trust that such a class of molecular rotors promises new applications in the quantitation of biological processes.

## Data Availability

The raw data supporting the conclusion of this article will be made available by the authors without undue reservation.
