# Supplementary figures and images for: A Fluorogenic Far Red-Emitting Molecular Viscometer for Ascertaining Lysosomal Stress in Live Cells and Caenorhabditis elegans
Source: Front Chem. 2022 Mar 11;10:840297. doi: 10.3389/fchem.2022.840297 (PMC8961804; doi:10.3389/fchem.2022.840297)

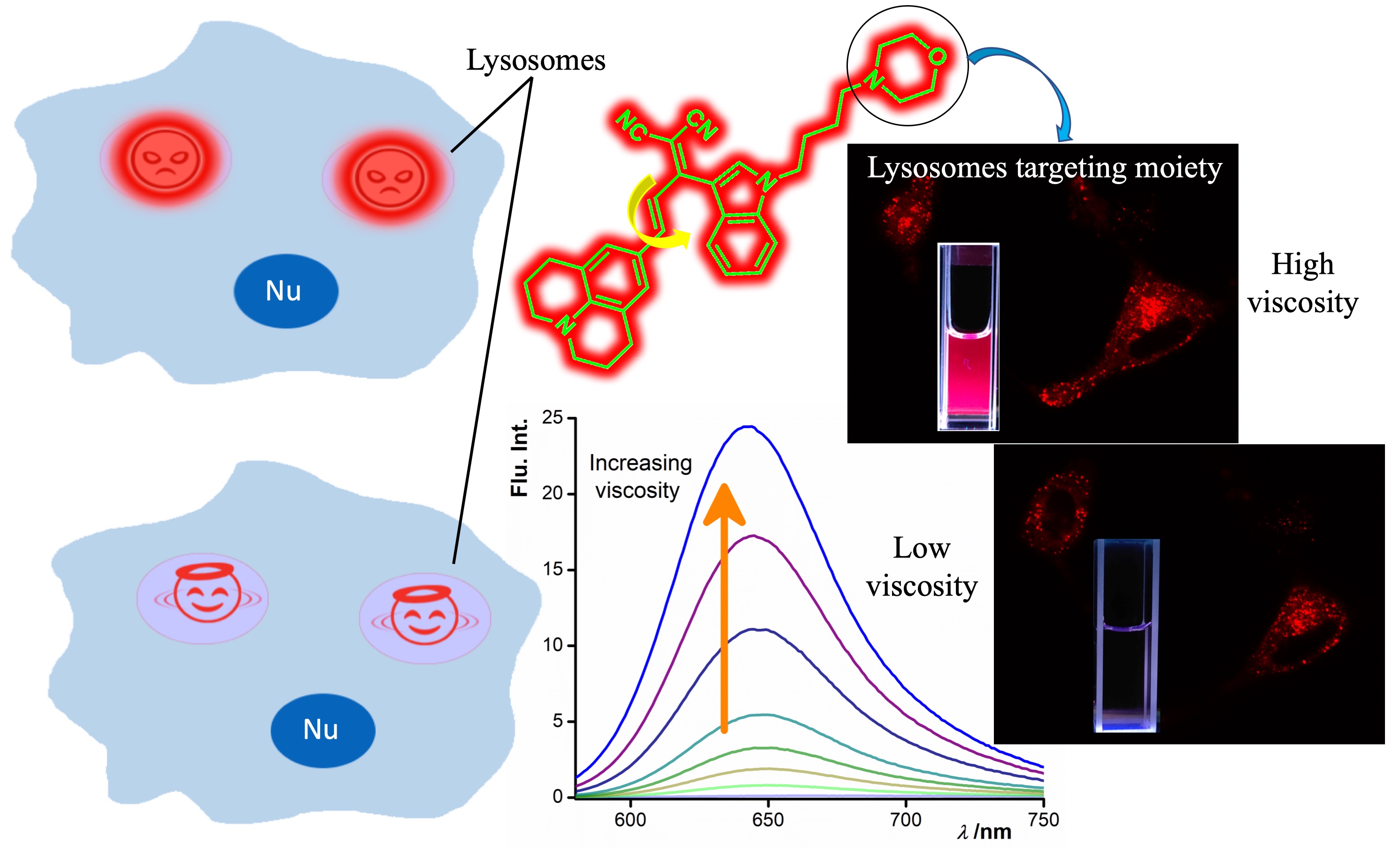

Supplement: Supplementary file 1 [file Image1.JPEG]
