# Supplementary material for: A Fluorogenic Far Red-Emitting Molecular Viscometer for Ascertaining Lysosomal Stress in Live Cells and Caenorhabditis elegans
Source: Front Chem. 2022 Mar 11;10:840297. doi: 10.3389/fchem.2022.840297 (PMC8961804; doi:10.3389/fchem.2022.840297)
Supplement: Supplementary file 2 [file DataSheet1.pdf]

Supporting Information (SI)

A Fluorogenic Far Red-Emitting Molecular Viscometer for Ascertaining Lysosomal Stress in Live Cells and Caenorhabditis elegans

Akshay Silswal,[a] Ashutosh Kanojiya [a] and Apurba Lal Koner *[a]

[a] *Bionanotechnology Lab, Department of Chemistry Indian Institute of Science Education and Research Bhopal, Bhopal Bypass Road, Bhauri, Bhopal, Madhya Pradesh, INDIA,* E-mail*:* akoner@iiserb.ac.in

Table of Content

1. Experimental Section

1.1 Synthetic Scheme

1.1.1 Synthesis of compound **1**……………………………………………………3

1.1.2 Synthesis of compound **2**…………………………………………………....3-4

1.1.3 Synthesis of compound **DCAJ**…………………………………………..…4-5

1.1.4 Synthesis of compound **3**……………………………………………...……..5

1.1.5 Synthesis of compound **JIND**…………………………..................……....5-6

1.1.6 Synthesis of compound **4**…………………………...…………………..……6-7

1.1.7 Synthesis of compound **JIND-Mor**…………………..………………………..7-8

1.2. Characterization by NMR and Mass

Fig. S1: ^{1}H NMR of compound **1**……………………………………………………..9

Fig. S2: HRMS of compound **1**………………………..............................………..…..9

Fig. S3: ^{1}H NMR of compound **2**……………………………………………..…..…10

Fig. S4: HRMS of compound **2**……………………………………………......…...10

Fig. S5: ^{1}H NMR of compound **3**……………………………………………...…...…11

Fig. S6: HRMS of compound **3**…………………………………………………....…...11

Fig. S7: ^{1}H NMR of compound **DCAJ**…………………………………………..……..12

Fig. S8: ^{13}C NMR of compound **DCAJ**………………….……………………………12

Fig. S9: HRMS of compound **DCAJ**……………………………………………..…………….13

Fig. S10: ^{1}H NMR of compound **JIND**…………..…………………….…….…..…………13

Fig. S11: ^{13}C NMR of compound **JIND**…………………………………………………..14

Fig. S12: HRMS of compound **JIND**………………………………….……………….14

Fig. S13: ^{1}H NMR of compound **4**…………………………………….……………....15

Fig. S14: HRMS of compound **4**……………………………….…….….………………15

Fig. S15: ^{1}H NMR of compound **JIND-Mor**…………………………..…………………16

Fig. S16: ^{13}C NMR of compound **JIND-Mor**……………………………………………16

Fig. S17: HRMS of compound **JIND-Mor**…………………….……………….…..…17

1.3. Photophysical Properties, FMO, cytotoxicity, and Live-cell imaging

Fig. S18: Optical purity of compounds…………………………..………………..…17

Fig. S19: Validation of Beer-Lambert law of **JIND**…………………..………………..18

Fig. S20: Solvent dependent UV-Vis. and fluorescence spectra…………..……………...18

Measurement of Relative quantum yield……………………………………………………..19

Table S1: Photophysical parameters of molecular viscometers………….…...…………19

Fig. S21: FMO pictures of **JIND-Mor**…………………………..……………………..20

Fig. S22: Thermostability of **JIND** in EG……………………….……………………21

Fig. S23: Photostability of the compounds……………………..………………………21

Fig. S24: pH stability of the compounds………………...……………………………22

Fig. S25: Plot of emission maxima with the $E_T(30)$ parameter……………..………………23

Fig. S26: Fluorescence properties of **JIND** in different of glycerol methanol mixture...……24

Fig. S27: MTT assay of **JIND-Mor**…………………………………….………………..25

Fig. **S28**: Colocalization of **JIND-Mor** in U-87 MG cell lines…………..………………25

Table S2: Reported molecular viscometers for lysosomal viscosity…….……..…....…...26-27

1. Experimental Section

1.1 Synthetic Scheme:

1.1.1 2,3,6,7-tetrahydro-1H,5H-pyrido[3,2,1-ij]quinoline-9-carbaldehyde (1):

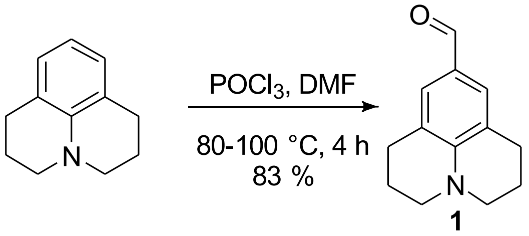

Synthetic Procedure:

$POCl_3$ (596 μL, 6.38 mmol) was added dropwise to anhydrous DMF (1.5 mL, 19 mmol) cooled in ice-bath under nitrogen atmosphere. The mixture was stirred at room temperature for 30 min and then transferred to a round bottom flask containing Julolidine (1 g, 5.8 mmol) and the resulting mixture was heated at 90 °C for 4 h. After cooling, water was added to the reaction mixture and the mixture was neutralized with sodium bicarbonate. The mixture was then extracted with ethyl acetate and the organic layer was washed with brine, dried with Na_2SO_4, filtered and concentrated in vacuum to afford compound (0.97 mg) as greenish solid with 83% yield.

^{1}H NMR (400 MHz, $CDCl_3$) δ 9.56 (s, 1H), 7.26 (s, 2H), 3.26 (t, 4H), 2.73 (t, J = 6.3 Hz, 4H), 1.97 – 1.88 (m, 4H). HRMS (ESI) m/z $[M+Na]^+$ calculated mass- 224.1046, obtained mass- 224.1065

1.1.2 2-(1-(p-tolyl)ethylidene)malononitrile (2):

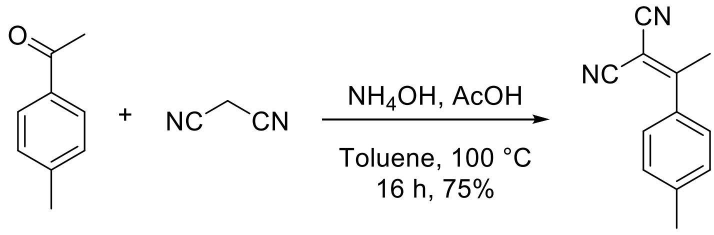

Synthetic Procedure:

To the mixture of 4-methylacetophenone (668 μL, 5 mmol) and malononitrile (363 mg, 5.5 mmol) in toluene, ammonium acetate (424 mg, 5.5 mmol) and acetic acid (860 μL, 15 mmol) was added in a round bottom flask. The round bottom flask is then connected with Dean-Stark apparatus and refluxed for 16 h. Then reaction mixture was cooled down and washed with ammonium chloride solution and brine, dried with Na_2SO_4, filtered and concentrated in vacuum to get red solid crude which was purified by column chromatography using silica gel and 2-5 % ethyl acetate/hexane as eluent to obtain pure product with 75 % yield.

^{1}H NMR (500 MHz, $CDCl_3$) δ 7.51 (d, J = 8.3 Hz, 2H), 7.33 (d, J = 8.1 Hz, 2H), 2.65 (s, 3H), 2.45 (s, 3H). HRMS (ESI) m/z $[M+H]^+$ calculated mass- 183.0917, obtained mass- 183.0914

1.1.3 (E)-2-(3-(2,3,6,7-tetrahydro-1H,5H-pyrido[3,2,1-ij]quinolin-9-yl)-1-(p-tolyl)allylidene)malononitrile (DCAJ):

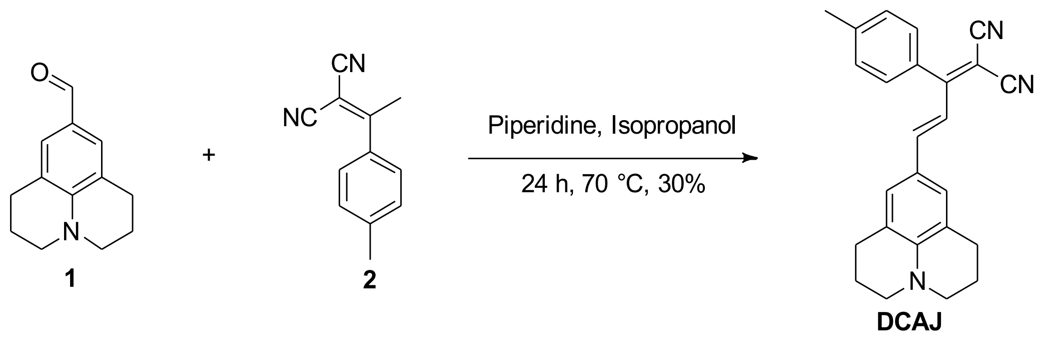

Synthetic Procedure:

To the compound **2** (166 mg, 0.9 mmol) in isopropanol, piperidine (90 μl, 0.9 mmol) was added dropwise. After stirring the solution for 1 h at room temperature, compound **1** (202 mg, 1 mmol) was added to it and the solution was kept on heating at 70 °C for 24 h. After cooling, on filtration violet solid compound was obtained which further purified by recrystallization from hot ethanol to obtain the product with 30 % yield.

^{1}H NMR (500 MHz, DMSO-D_6) δ 7.38 (d, J = 7.9 Hz, 2H), 7.30 (d, J = 8.1 Hz, 2H), 7.15 (d, J = 14.9 Hz, 1H), 7.08 (s, 2H), 6.70 (d, J = 14.9 Hz, 1H), 3.32 – 3.27 (m, 4H), 2.66 (t, J = 6.2 Hz, 4H), 2.41 (s, 3H), 1.87 – 1.80 (m, 4H). ^{13}C NMR (101 MHz, $CDCl_3$) δ 171.65, 150.44, 146.56, 140.75, 131.32, 129.39, 129.01, 121.41, 121.25, 117.96, 115.30, 114.71, 74.79, 50.10,

27.56, 21.49, 21.29. HRMS (APCI) m/z $[M+H]^+$ calculated mass- 366.1965, obtained mass- 366.1976

1.1.4 2-(1-(1H-indol-3-yl)ethylidene)malononitrile (3):

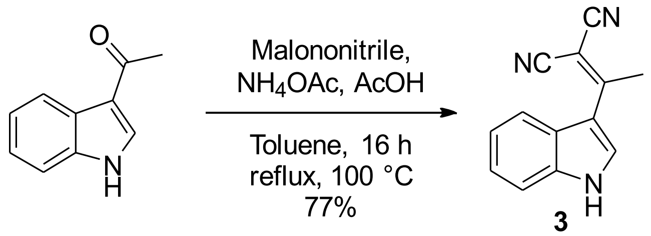

Synthetic Procedure:

3-Acetylindole (500 mg, 3.14 mmol) and malononitrile (228 mg, 3.45 mmol) in toluene was taken in round bottom flask. Ammonium acetate (266 mg, 77 mmol) was dissolved in acetic acid (1.6 ml, 28.26 mmol) and added dropwise to round bottom flask. Then the solution is refluxed for 16 h. After completion of reaction toluene is evaporated. To the mixture, ethyl acetate was added and washed three times with water followed by brine, dried in sodium sulphate anhydrous, and solvent is removed under vacuum. Product is then purified by column chromatography using silica gel and 10-20 % ethyl acetate/hexane as eluent to give white solid with 77 % yield.

^{1}H NMR (400 MHz, DMSO-D_6) δ 12.50 (s, 1H), 8.40 (d, J = 3.2 Hz, 1H), 7.81 (d, J = 7.6 Hz, 1H), 7.57 (d, J = 8.2 Hz, 1H), 7.38 – 7.18 (m, 2H), 2.78 (s, 3H). HRMS (ESI) m/z $[M+H]^+$ calculated mass- 208.0869, obtained mass- 208.0847

1.1.5 (E)-2-(1-(1H-indol-3-yl)-3-(2,3,6,7-tetrahydro-1H,5H-pyrido[3,2,1-ij]quinolin-9-yl)allylidene)malononitrile (JIND):

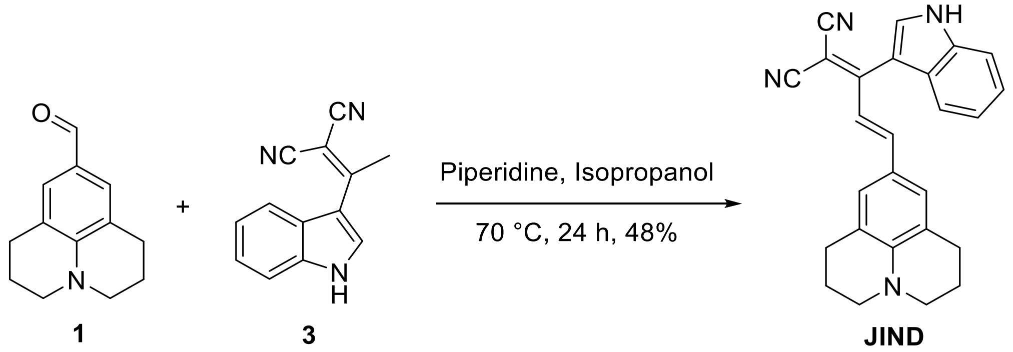

Synthetic Procedure:

To the compound **3** (700 mg, 3.37 mmol) in isopropanol, piperidine (334 μl, 3.38 mmol) was added dropwise. After stirring the solution for 1 h at room temperature, compound **1** (748 mg, 3.72 mmol) was added to it and the solution was kept on heating at 70 °C for 24 h. Reaction mixture was then cool down and filtered using cold isopropanol. Residue was collected and further purified by precipitation using ethyl acetate and hexane to give the blood red solid product with 48% yield.

^{1}H NMR (500 MHz DMSO-d$_6$) δ 12.14 (s, 1H), 7.92 (s, 1H), 7.55 (d, 1H), 7.44 (d, 1H), 7.23 (m, 2H), 7.14 (t, 1H), 7.07 (m, 3H), 3.29 (t, 4H), 2.67 (t, 4H), 1.85 (m, 4H). ^{13}C NMR (126 MHz, DMSO-d$_6$) δ 164.97, 150.40, 146.67, 137.16, 131.26, 129.27, 126.04, 122.99, 121.43, 121.21, 120.65, 117.22, 116.83, 116.50, 113.02, 109.71, 69.40, 49.85, 27.40, 21.24. HRMS (ESI) m/z [M]$^+$ calculated mass- 390.1839 obtained mass- 390.1809

1.1.6 (E)-2-(1-(1-(4-bromobutyl)-1H-indol-3-yl)-3-(2,3,6,7-tetrahydro-1H,5H-pyrido[3,2,1-ij]quinolin-9-yl)allylidene)malononitrile (4):

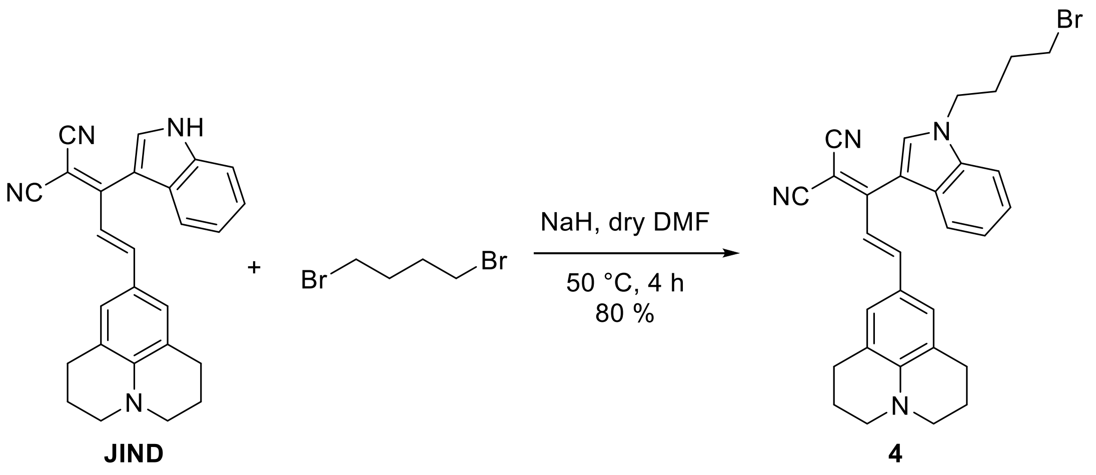

Synthetic Procedure:

Sodium hydride (6.75 mg, 0.28 mmol, 60% in oil) was washed with hexane and then kept in vacuum and dissolved in 1 mL dry DMF and then added to the compound **3** (100 mg, 0.26 mmol) dissolved in dry DMF (3 mL) dropwise and stirred for 30 mins at room temperature under N_2 atmosphere. Then dibromobutane (92.35 μL, 0.77 mmol) was added dropwise to reaction mixture and kept on stirring for 4 h at 50 °C. After completion, reaction mixture was dissolved in ice cold water and compound is extracted using ethyl acetate (3x20 mL). Combine organic phase was washed with brine and dried over anhydrous sodium sulphate. Then ethyl acetate was evaporated and product is collected. The blood red solid product was further purified using neutral alumina gel and 20 % ethyl acetate/hexane and 1% triethyl amine as eluent with 80% yield.

^{1}H NMR (500 MHz, DMSO-D$_6$) δ 7.94 (s, 1H), 7.65 (d, J = 8.2 Hz, 1H), 7.44 (d, J = 8.1 Hz, 1H), 7.28 (t, J = 7.7 Hz, 1H), 7.19 – 7.14 (m, 2H), 7.05 – 7.00 (m, J = 7.4 Hz, 3H), 4.34 (t, J = 6.7 Hz, 2H), 3.67-3.71(m, 4H), 3.28 – 3.23 (m, 4H), 2.66 – 2.60 (m, J = 6.0 Hz, 4H), 1.96 – 1.89 (m, 2H), 1.85 – 1.77 (m, 4H). HRMS (ESI) m/z [M+H]$^+$ calculated mass- 527.1630 obtained mass- 527.1626

1.1.7 (E)-2-(1-(1-(4-morpholinobutyl)-1H-indol-3-yl)-3-(2,3,6,7-tetrahydro-1H,5H-pyrido[3,2,1-ij]quinolin-9-yl)allylidene)malononitrile (JIND-Mor):

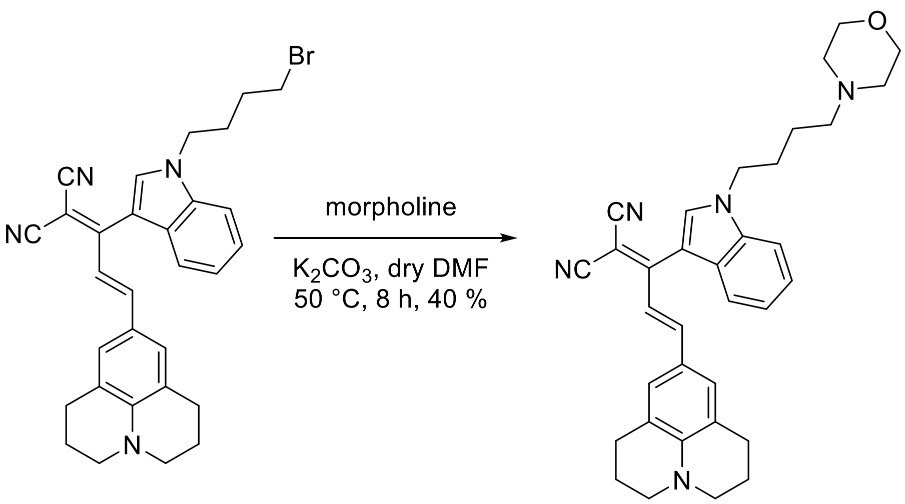

4

Synthetic Procedure:

Compound **4** (100 mg, 0.19 mmol) and potassium carbonate (53 mg, 0.38 mmol) was taken in a round bottom flask and kept on vacuum for 15 min and then dry DMF was added under N_2 atmosphere. To the reaction mixture, morpholine (165 μL, 1.9 mmol) was added and kept on heating for 8 h. After completion, reaction mixture was dissolved in ice cold water and compound is extracted using ethyl acetate (3x20 mL). Combine organic phase was washed with brine and dried over anhydrous sodium sulphate. Then solvent was evaporated and product was isolated using neutral alumina gel and 20 % ethyl acetate/hexane and 1% triethyl amine as eluent with 40% yield.

^{1}H NMR (400 MHz, $CDCl_3$) δ 7.60 (s, 1H), 7.55 (d, J = 8.0 Hz, 1H), 7.45 (d, J = 8.3 Hz, 1H), 7.37 – 7.30 (m, 2H), 7.18 (t, J = 7.5 Hz, 1H), 7.10 (d, J = 15.1 Hz, 1H), 7.00 (s, 2H), 4.27 (t, J = 7.0 Hz, 2H), 3.76 – 3.65 (m, 4H), 3.30 (t, J = 5.6 Hz, 4H), 2.73 (t, J = 6.2 Hz, 4H), 2.49 – 2.35 (m, 6H), 2.04 – 1.92 (m, 6H), 1.64 – 1.55 (m, 2H). ^{13}C NMR (101 MHz, $CDCl_3$) δ 164.40, 149.30, 146.17, 136.62, 131.57, 128.77, 127.09, 122.83, 121.49, 121.38, 121.32, 121.22, 117.27, 115.76, 110.21, 109.32, 71.90, 66.76, 58.06, 53.55, 50.07, 46.90, 30.93, 27.72, 27.58, 21.34. HRMS (ESI) m/z $[M+H]^+$ calculated mass- 532.3071 obtained mass- 532.3077

1.2 Characterization by NMR and Mass:

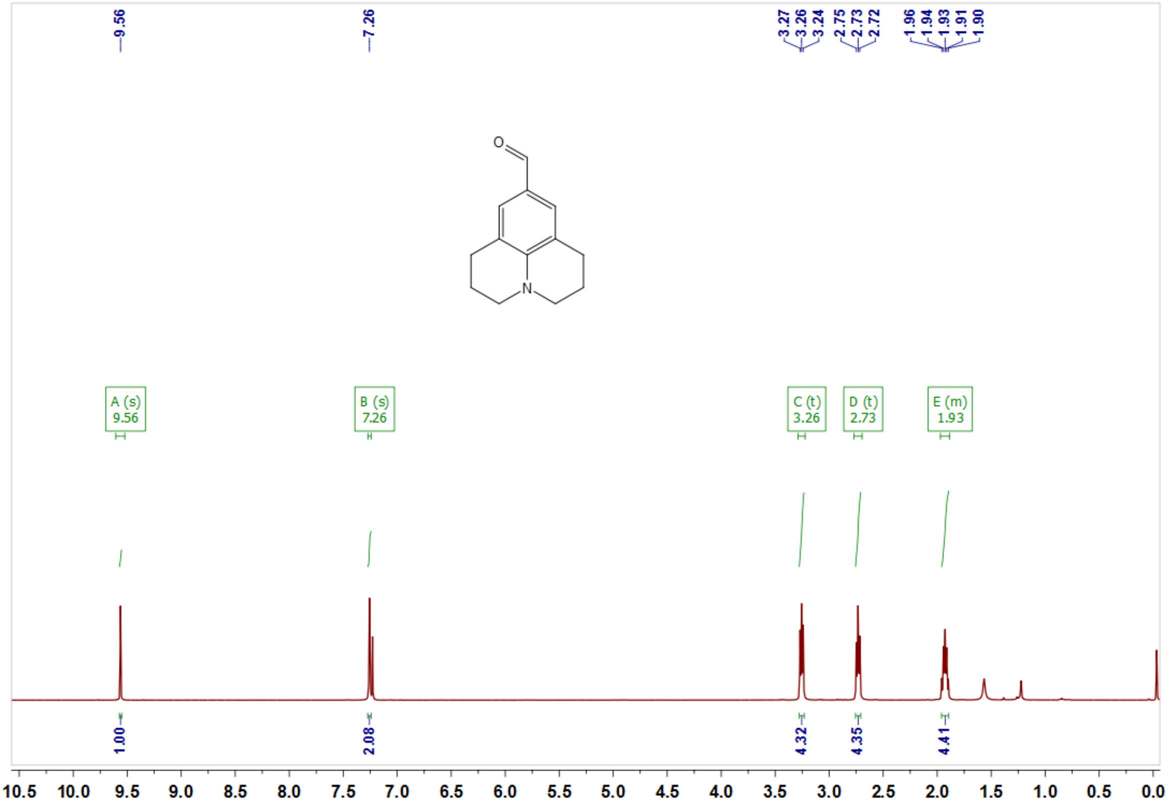

FIGURE S1: ^{1}H NMR spectrum of compound **1**.

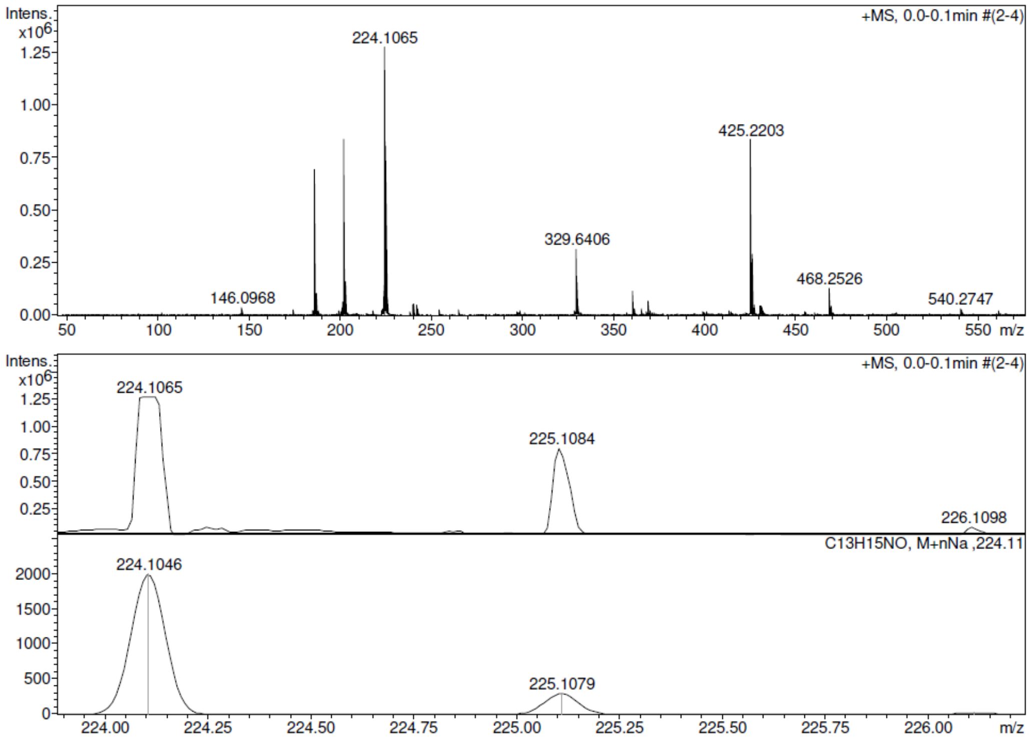

FIGURE S2: ESI-HRMS mass spectrum of compound **1**

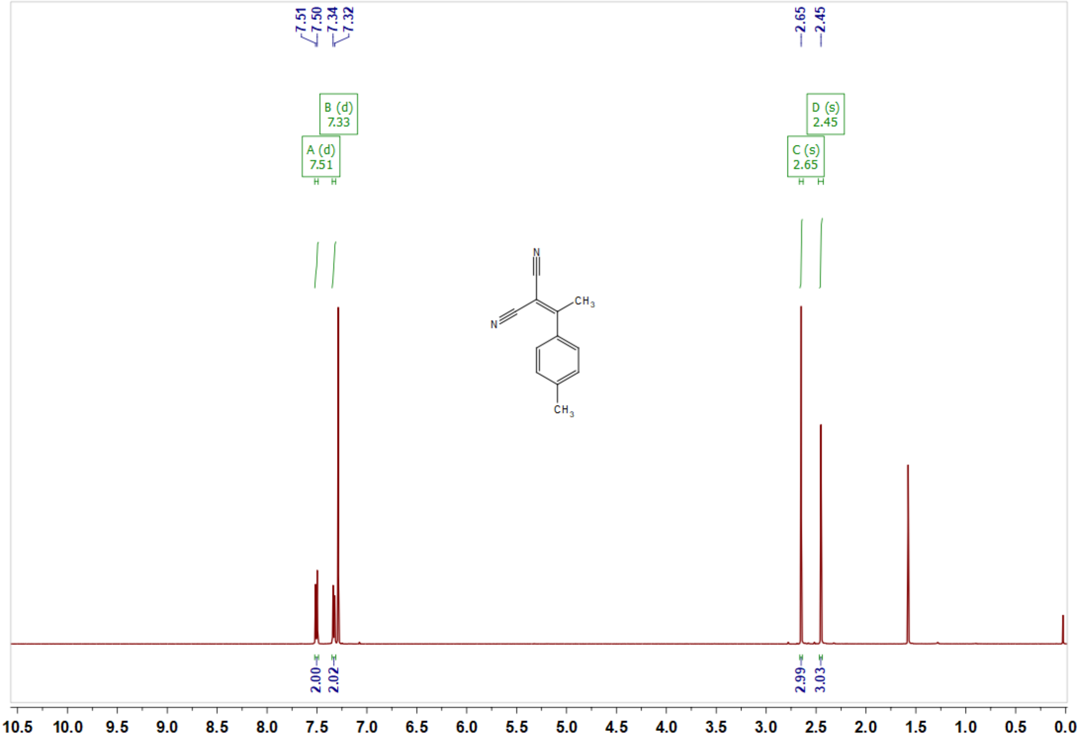

FIGURE S3: ^{1}H NMR spectrum of compound **2**.

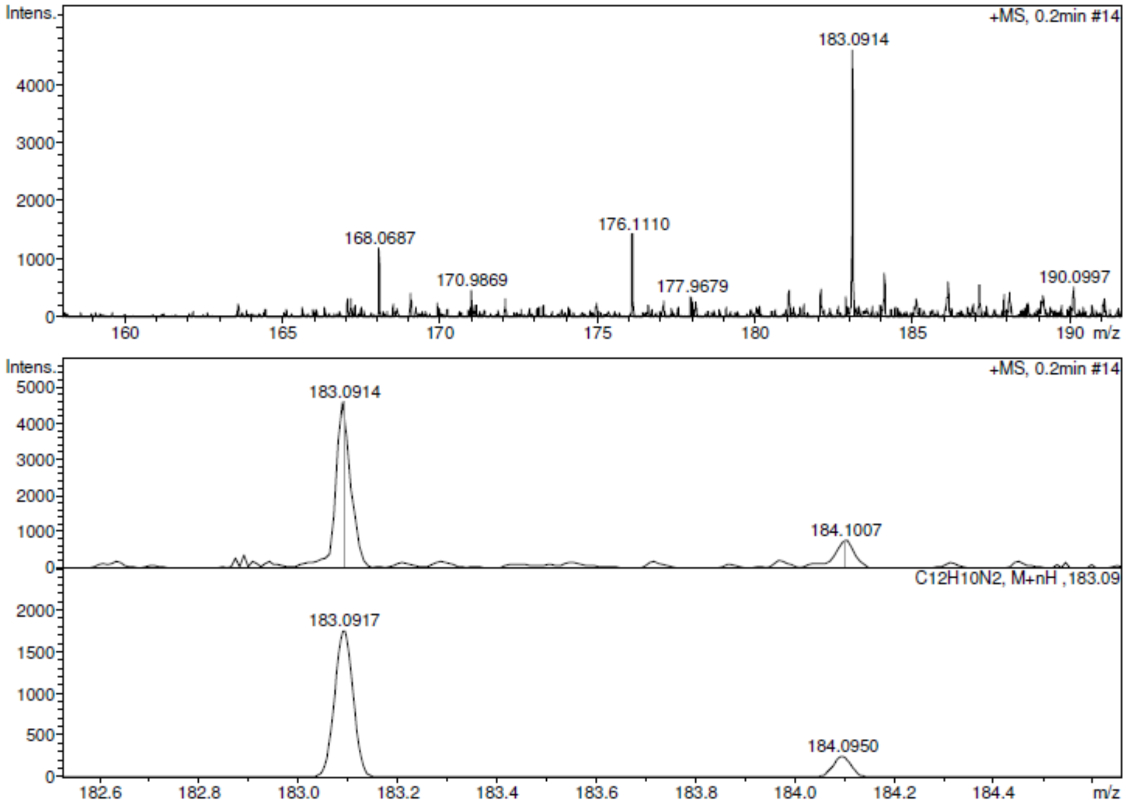

FIGURE S4: ESI-HRMS mass spectrum of compound **2**

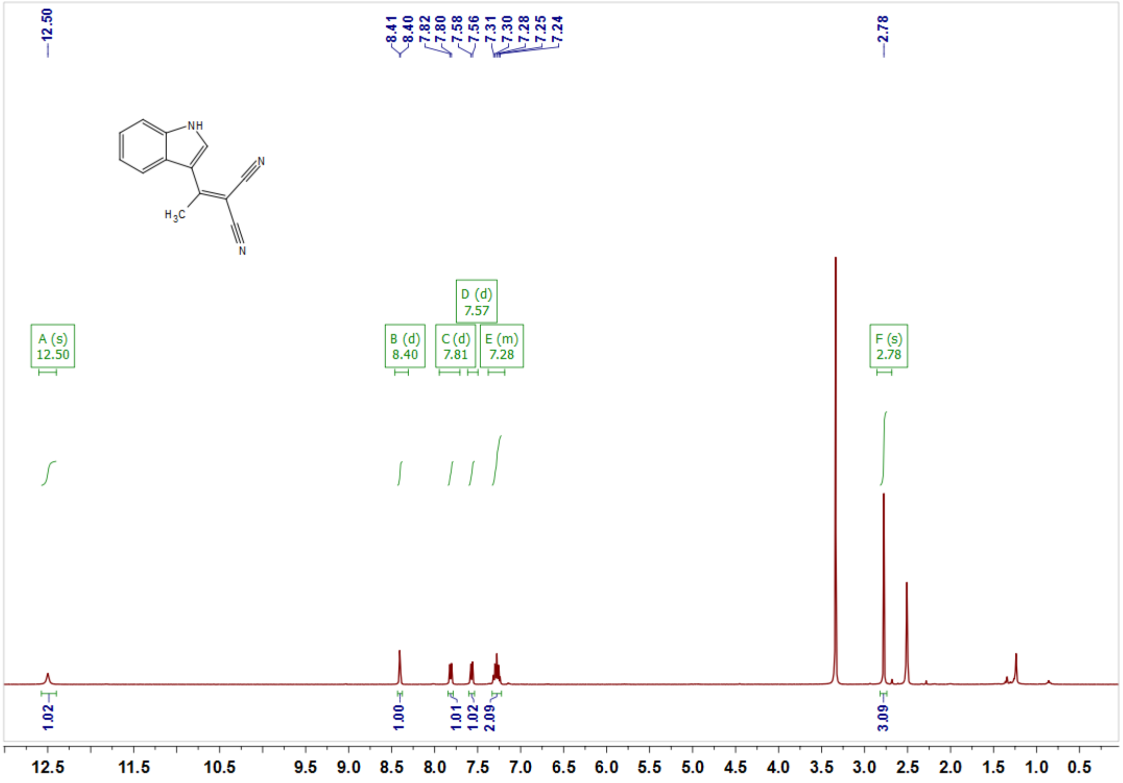

FIGURE S5: ^{1}H NMR spectrum of compound **3**.

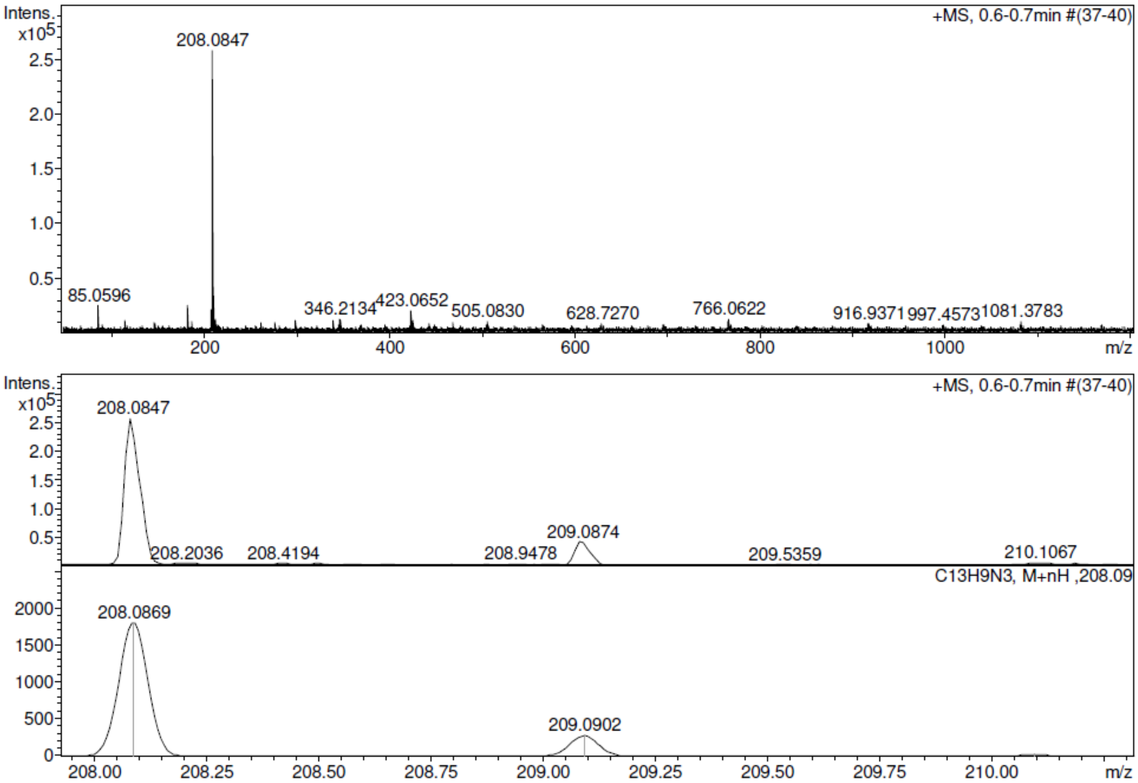

FIGURE S6: ESI-HRMS mass spectrum of compound **3**

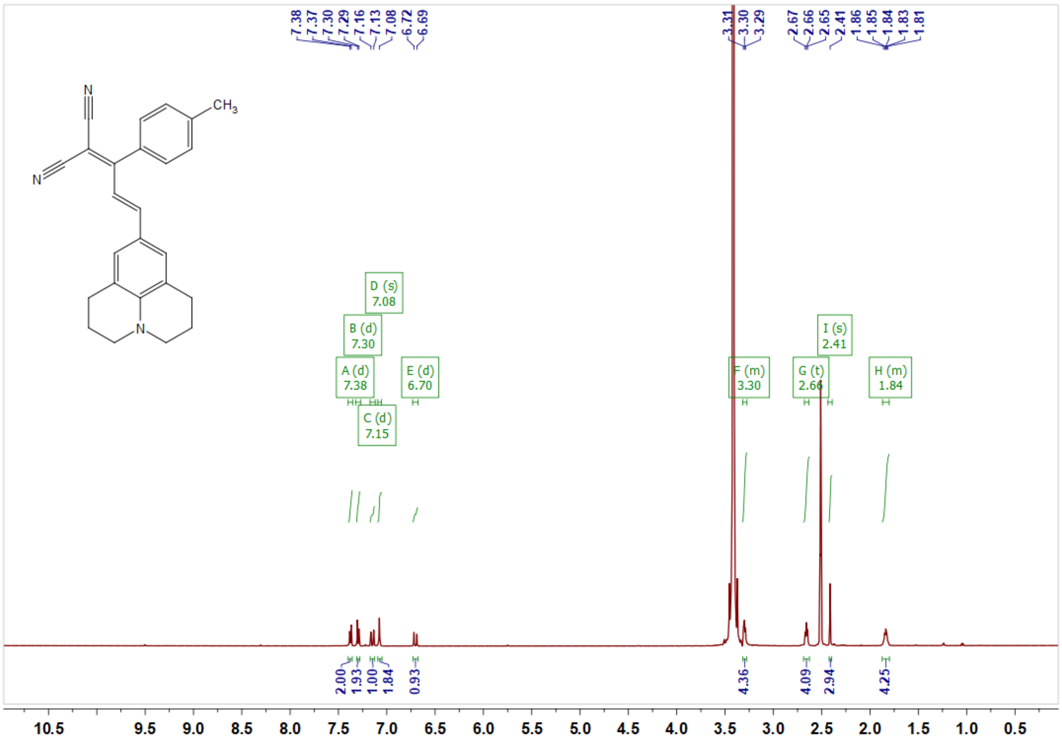

FIGURE S7: ^{1}H NMR spectrum of compound **DCAJ**.

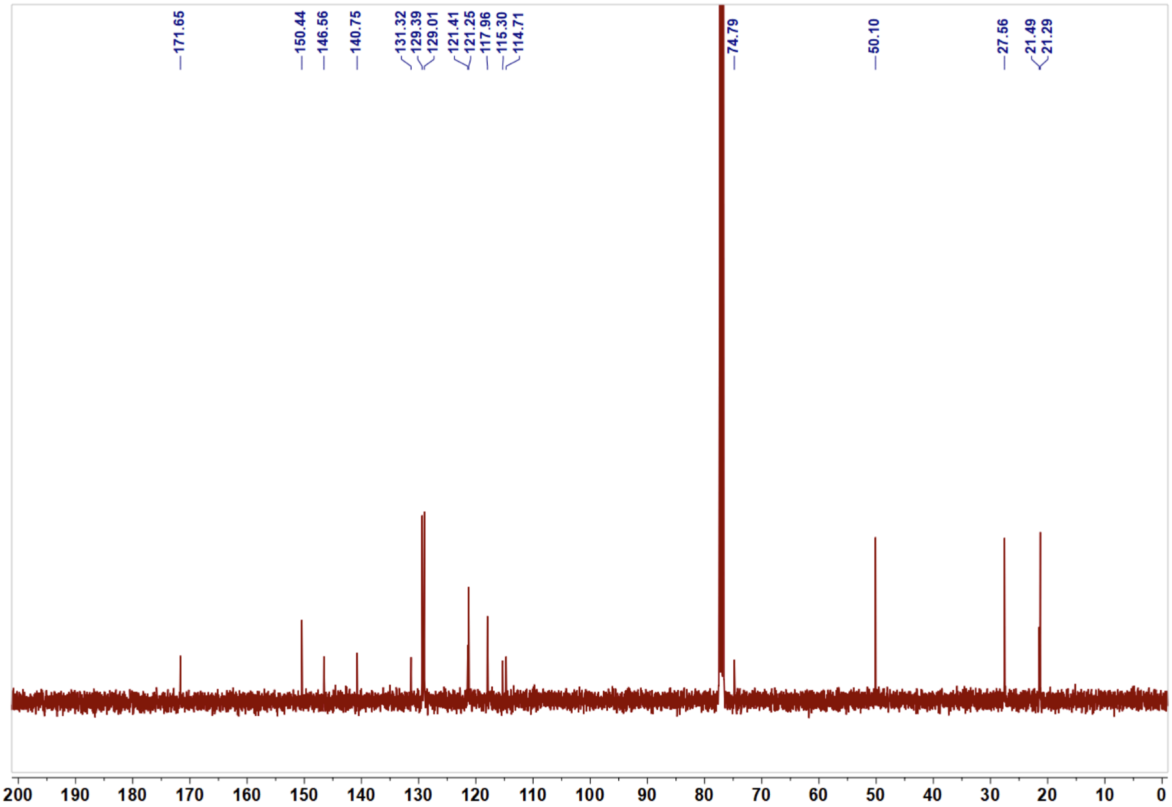

FIGURE S8: ^{13}C NMR spectrum of compound **DCAJ**.

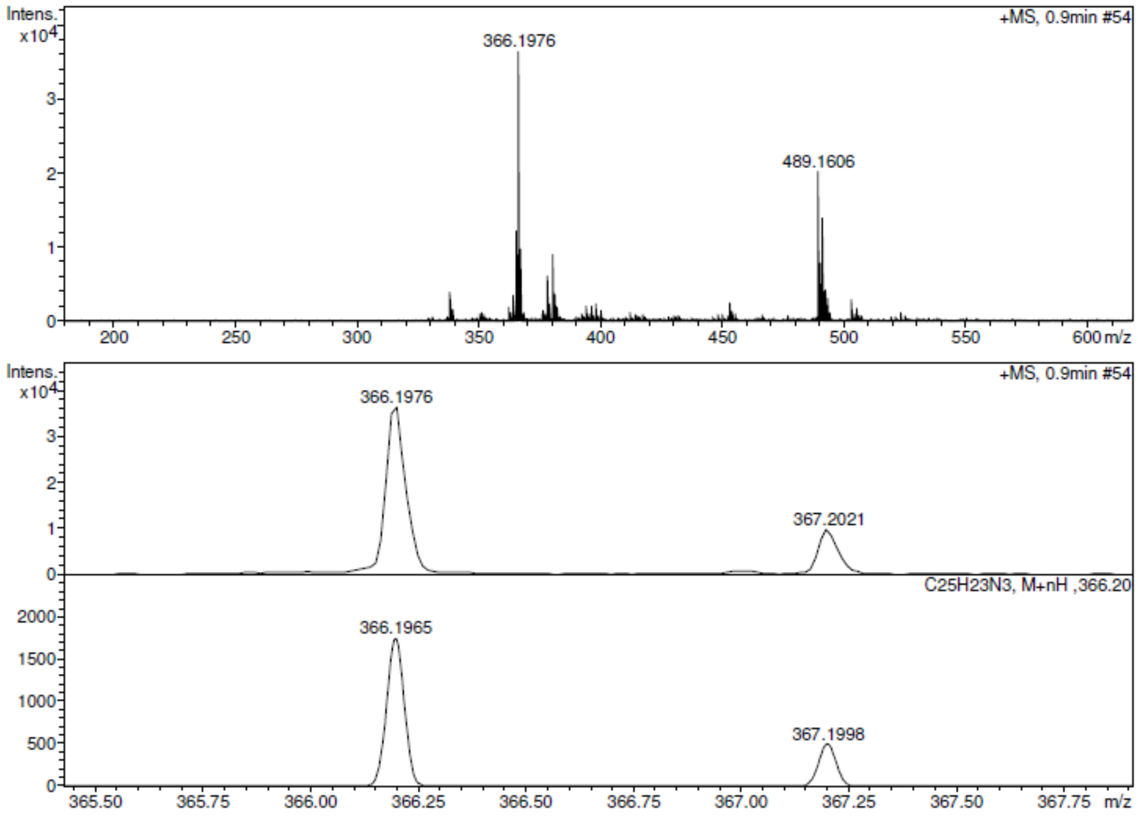

FIGURE S9: APCI-HRMS mass spectrum of compound **DCAJ**

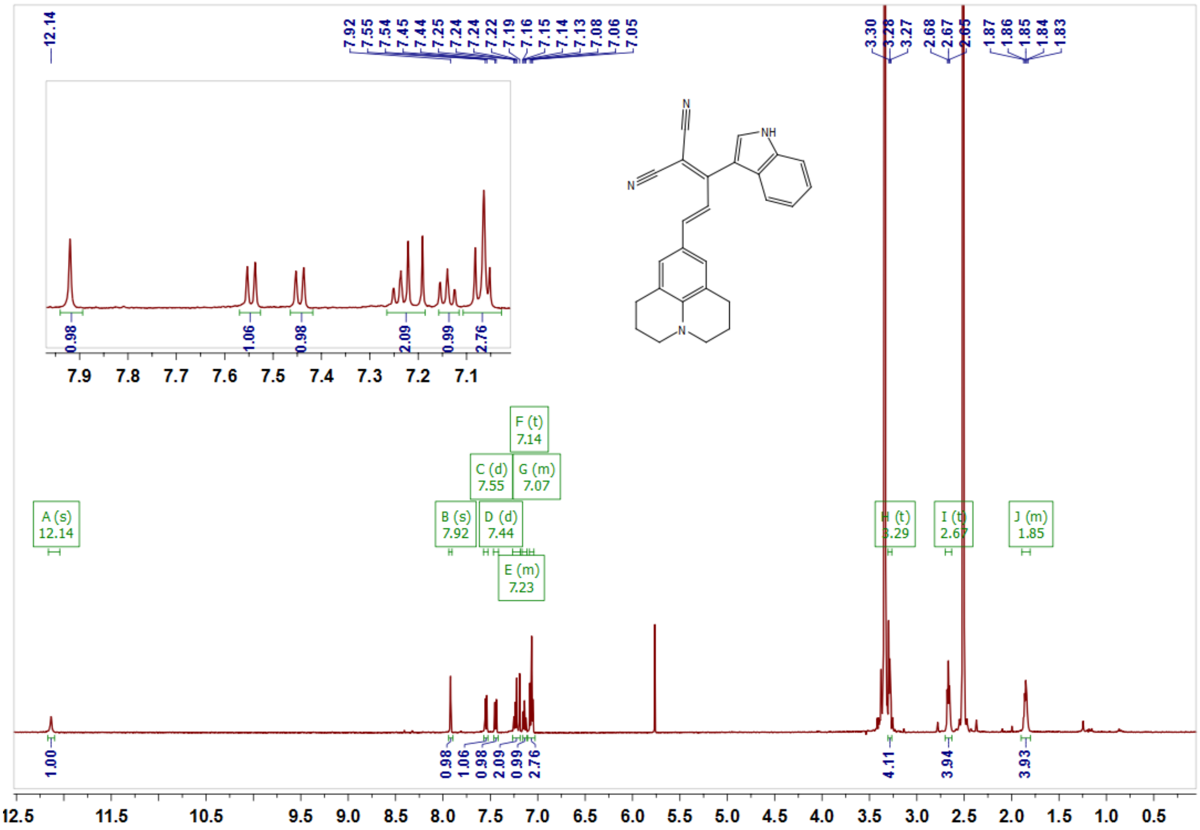

FIGURE S10: ^{1}H NMR spectrum of compound **JIND**.

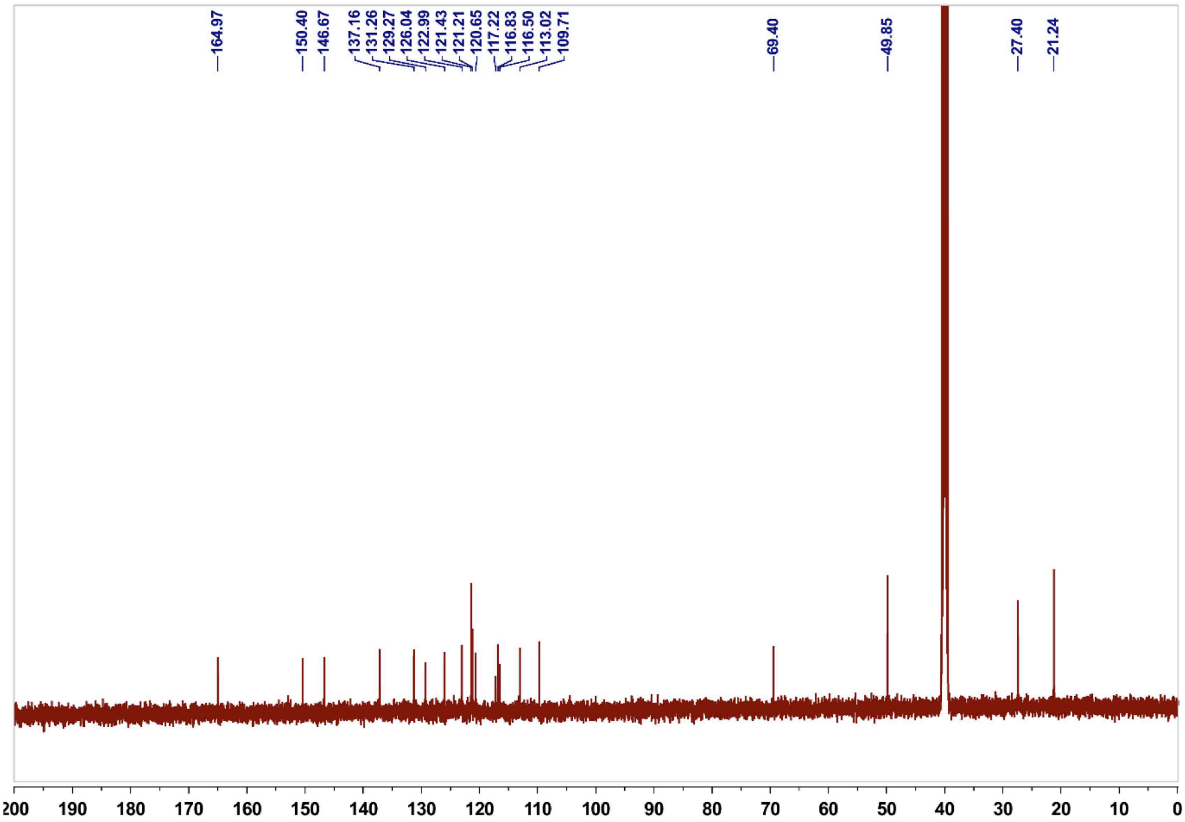

FIGURE S11: ^{13}C NMR spectrum of compound **JIND**.

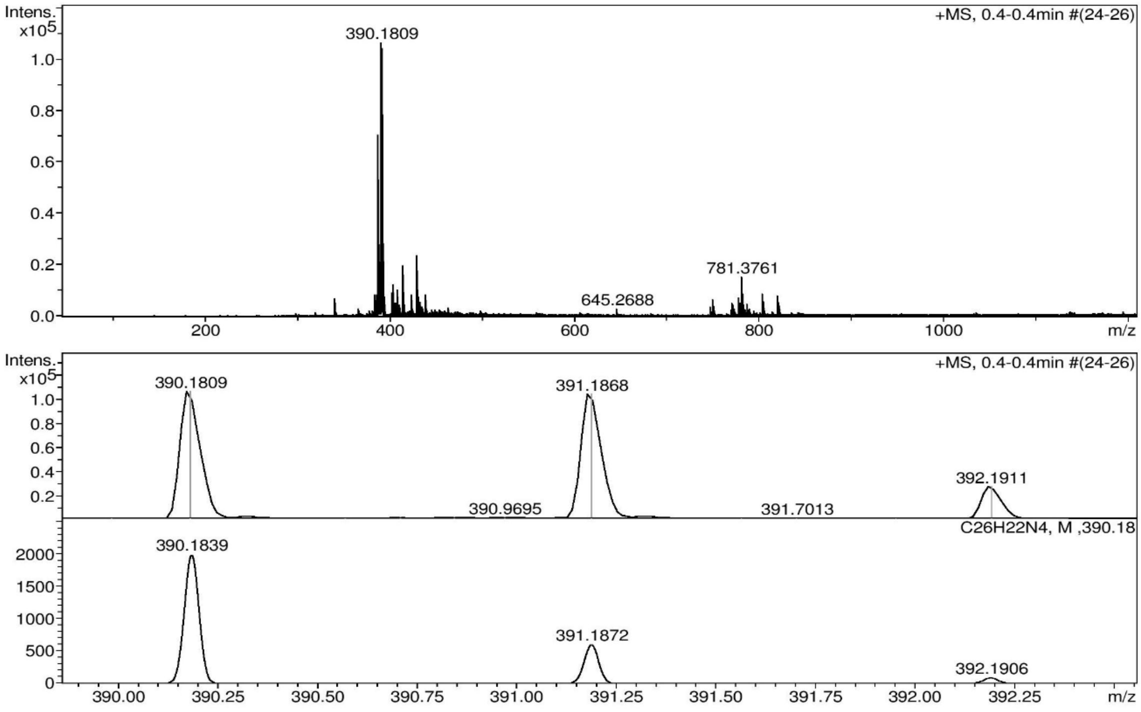

FIGURE S12: ESI-HRMS mass spectrum of compound **JIND**

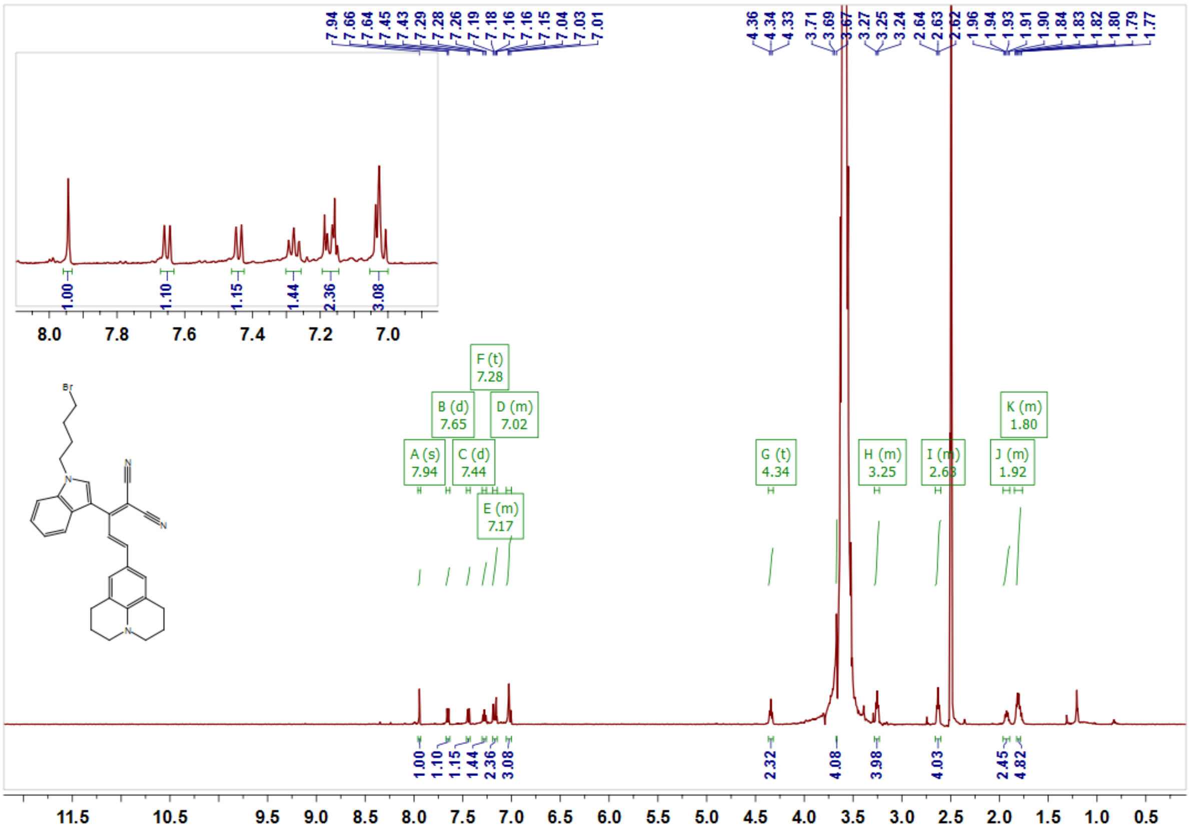

FIGURE S13: ^{1}H NMR spectrum of compound **4**.

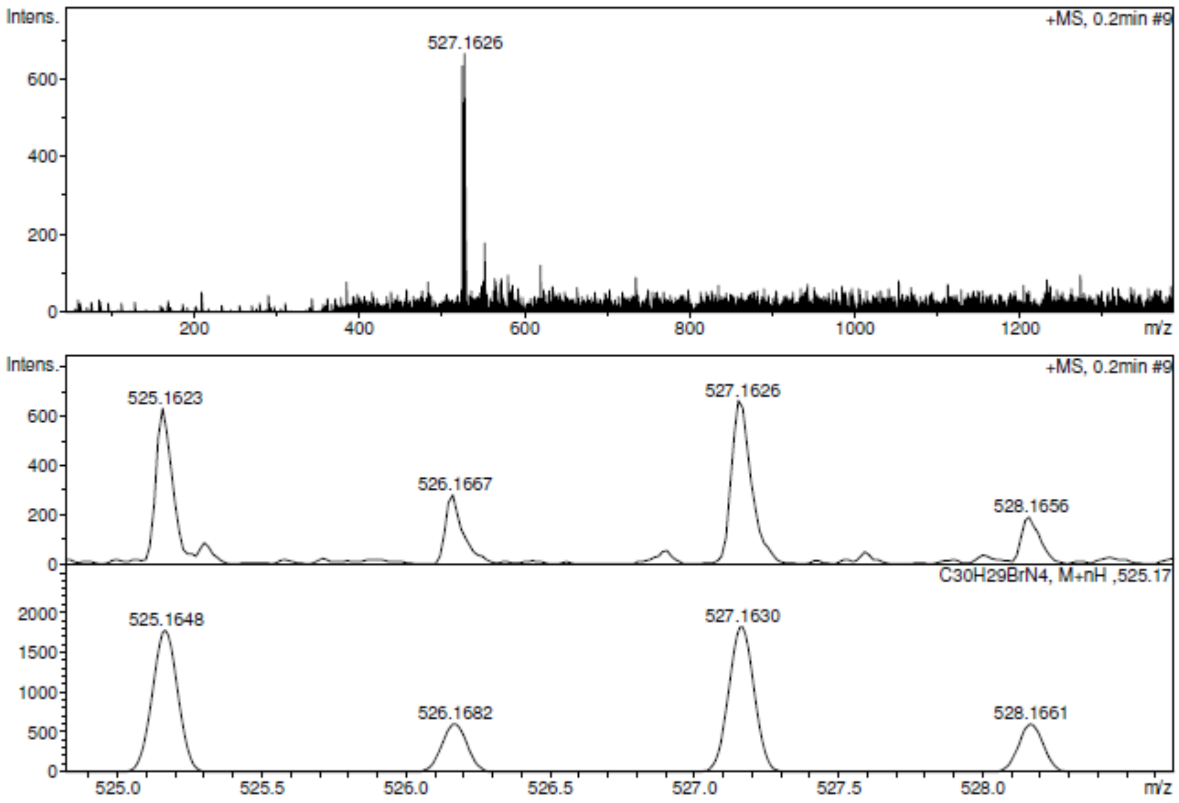

FIGURE S14: ESI-HRMS mass spectrum of compound **4**

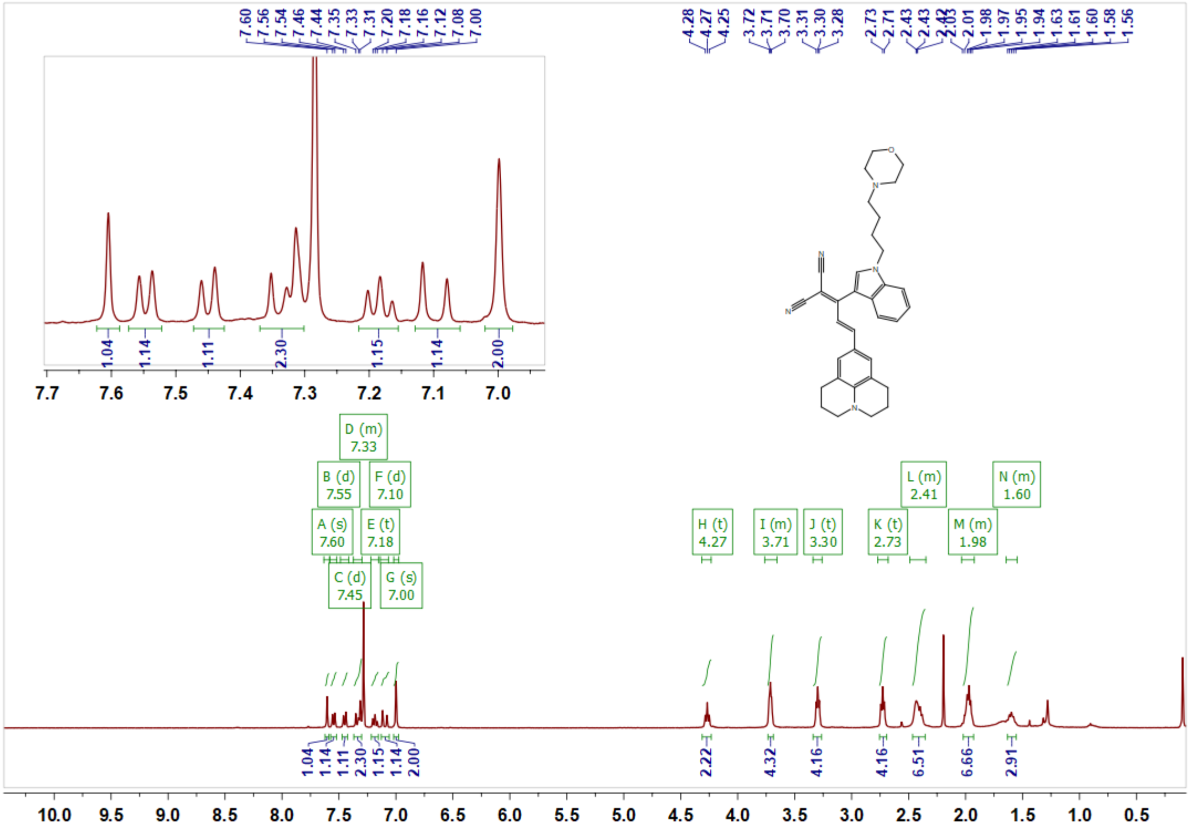

FIGURE S15: ^{1}H NMR spectrum of compound **JIND-Mor**.

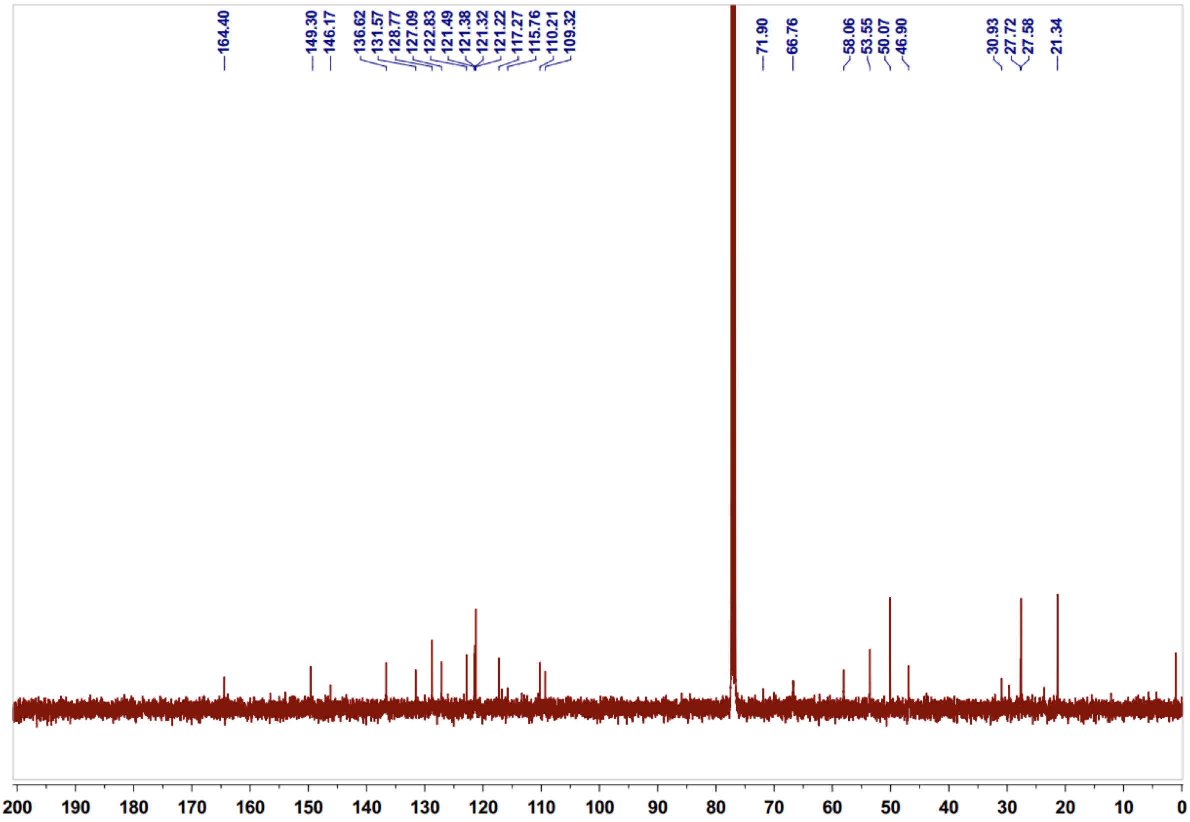

FIGURE S16: ^{13}C NMR spectrum of compound **JIND-Mor**.

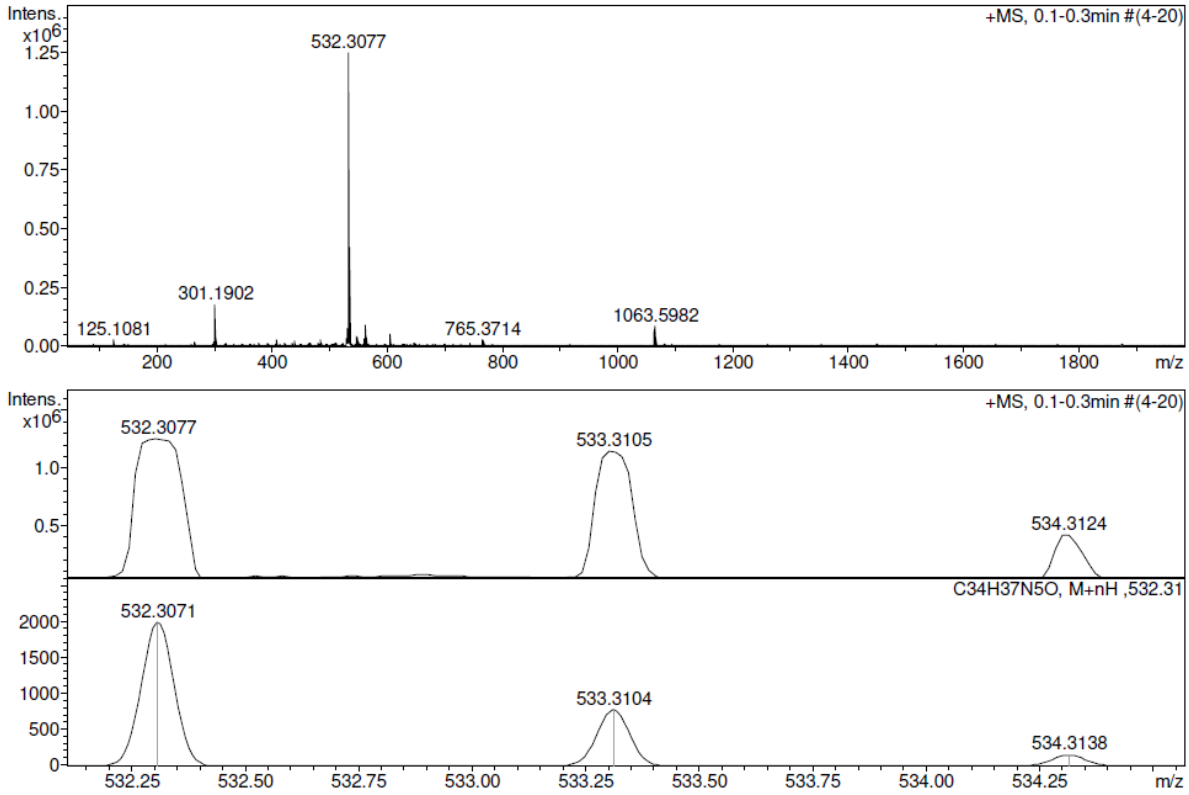

FIGURE S17: ESI-HRMS mass spectrum of compound **JIND-Mor**

1.3 Photophysical properties, FMO, cytotoxicity, and live-cell imaging:

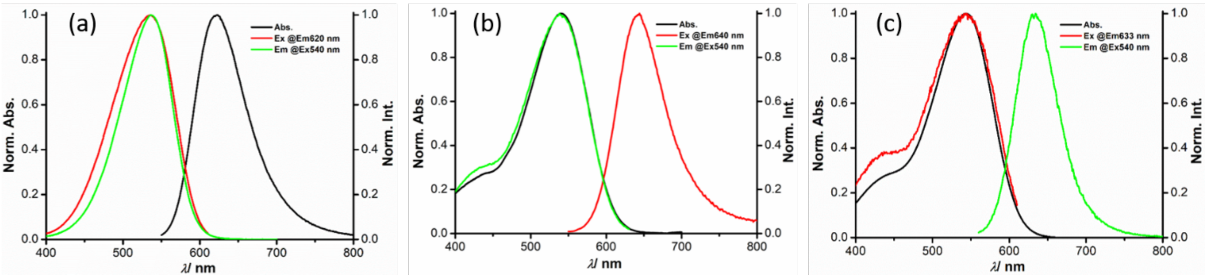

FIGURE S18: Absorption, excitation, and emission spectra to assess the optical purity of compound (a) **DCAJ** in DCM, (b) **JIND** in DMSO, and (c) **JIND-Mor**) in DMSO.

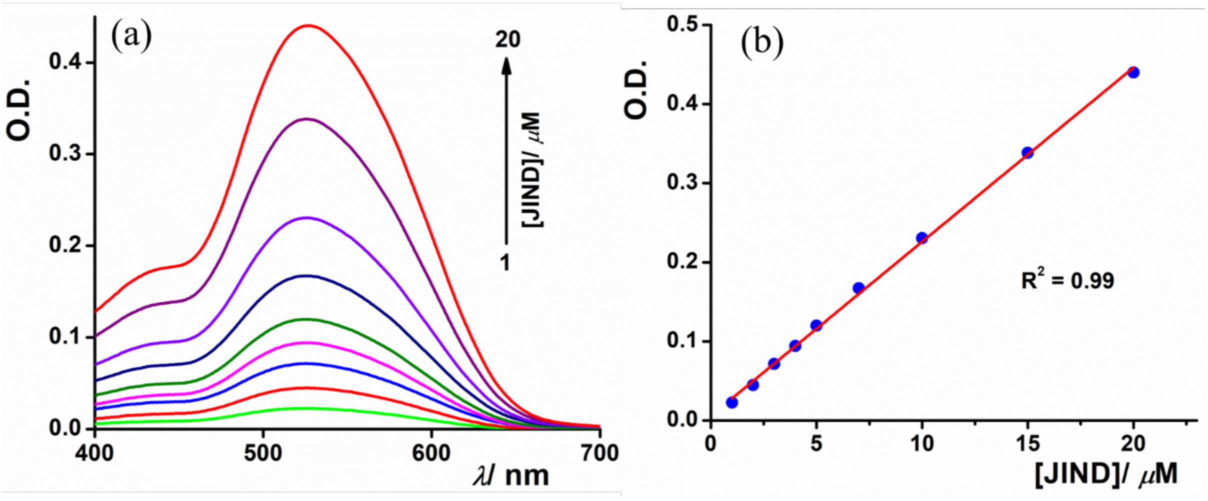

FIGURE S19: (a) Concentration dependent absorption spectrum of **JIND** in Water, (b) plot of **JIND** concentration vs. optical density (O.D.) at 525 nm of **JIND** obtained from (a), solid line shows the linear fitting. Beer-Lambert law is validated up to 20 μM.

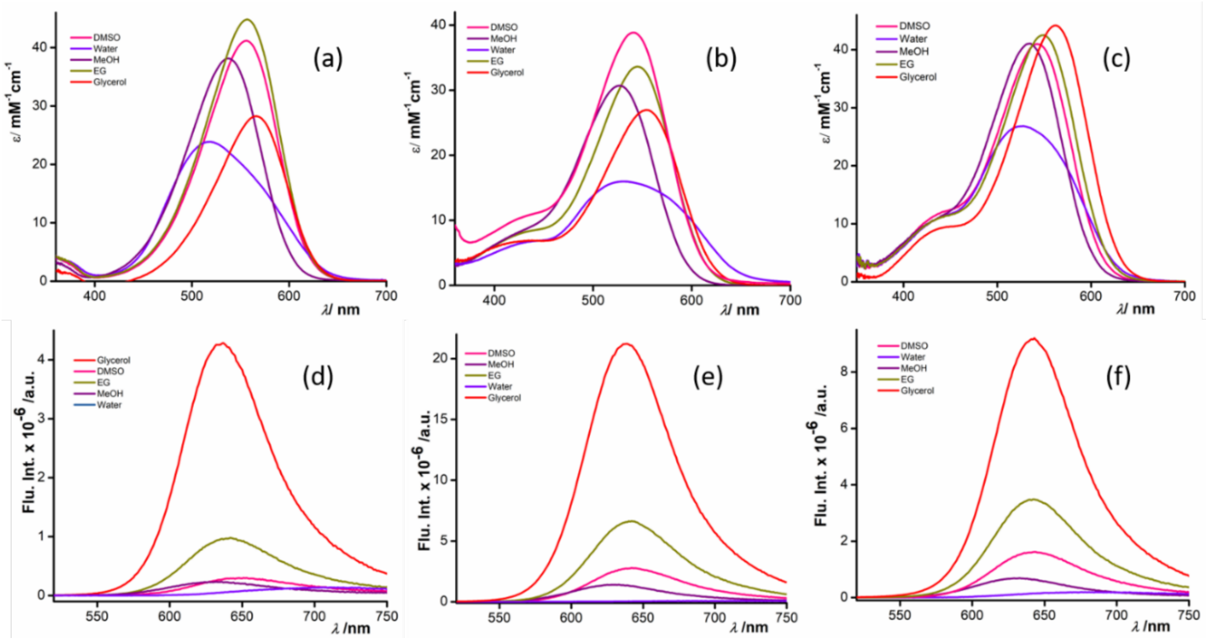

FIGURE S20: Solvent-dependent UV-Vis. absorption ((a) **DCAJ**, (b) **JIND**, and (c) **JIND-Mor**) and fluorescence properties ((d) **DCAJ**, (e) **JIND**, and (f) **JIND-Mor**).

Measurement of Relative quantum yield:

To measure the quantum yield of DCAJ, JIND and JIND-Mor in different solvents, Nile red (Quantum yield= 0.70 in dioxane) was used as standard. The following equation has been used for estimation of quantum yield:

$$\Phi = \Phi_R \times \frac{I}{I_R} \times \frac{1-10^{-OD_R}}{1-10^{-OD}} \times \frac{n^2}{n_R^2}$$

where n_R and n are the refractive indices of the solvents, OD_R and OD are the optical densities, Φ_R and Φ are the quantum yields, and I_R and I denote the integrated area of the fluorescence spectra of the reference and the sample of interest respectively.

Table S1: Photophysical parameters of Julolidine-based molecular viscometers

**Compound**	$\lambda_{max}^{abs.}$/ **nm**	$\lambda_{max}^{em.}$/ **nm**	**Stokes shift/ nm**	**Molar extinction coefficient/ $M^{-1}cm^{-1}$**	**Rel. QY× 10^{-3} [a]**	**Viscosity sensitivity (x)[b]**	
**DCAJ**	519	711	192	23000	0.4	0.35	Water
**JIND**	530	678	148	16000	0.4	0.49 (0.74)[e]	
**JIND-Mor**	528	663	135	27000	0.4	0.44	
**DCAJ**	557	642	87	44810	5.0	0.35	EG
**JIND**	545	642	97	33630	32.0	0.49	
**JIND-Mor**	548	643	95	42480	15.0	0.44	
**DCVJ**[c]	489	505	16	62900[d]	-	0.58	EG

a Relative Quantum Yield (QY) values were calculated using Nile Red in dioxane (QY=0.70) as a standard (https://www.photochemcad.com/compound-detail.php?name=Nile%20Red, *Anal. Biochem*, **1987**, *167* (2), 228-234)
b "x" represents the sensitivity of molecular rotor which is basically the slope of the line fitted into data points logτ over logη (water-glycerol mixture used to achieve different viscosities of solution). Where τ was in picosecond and η was in cP.
c calculated using logϕ Vs. logη equation (EG-glycerol mixture used to achieve different viscosities of solution, ref. Haidekker, M. A.; Ling, T.; Anglo, M.; Stevens, H. Y.; Frangos, J. A.; Theodorakis, E. A. *Chemistry & Biology* **2001,** *8* (2), 123-131.
d In Ethanol at 453 nm value obtained from Ref. Kung, C. E.; Reed, J. K., *Biochemistry* **1989,** *28* (16), 6678-6686.
e measured in methanol glycerol mixture from steady-state fluorescence measurement

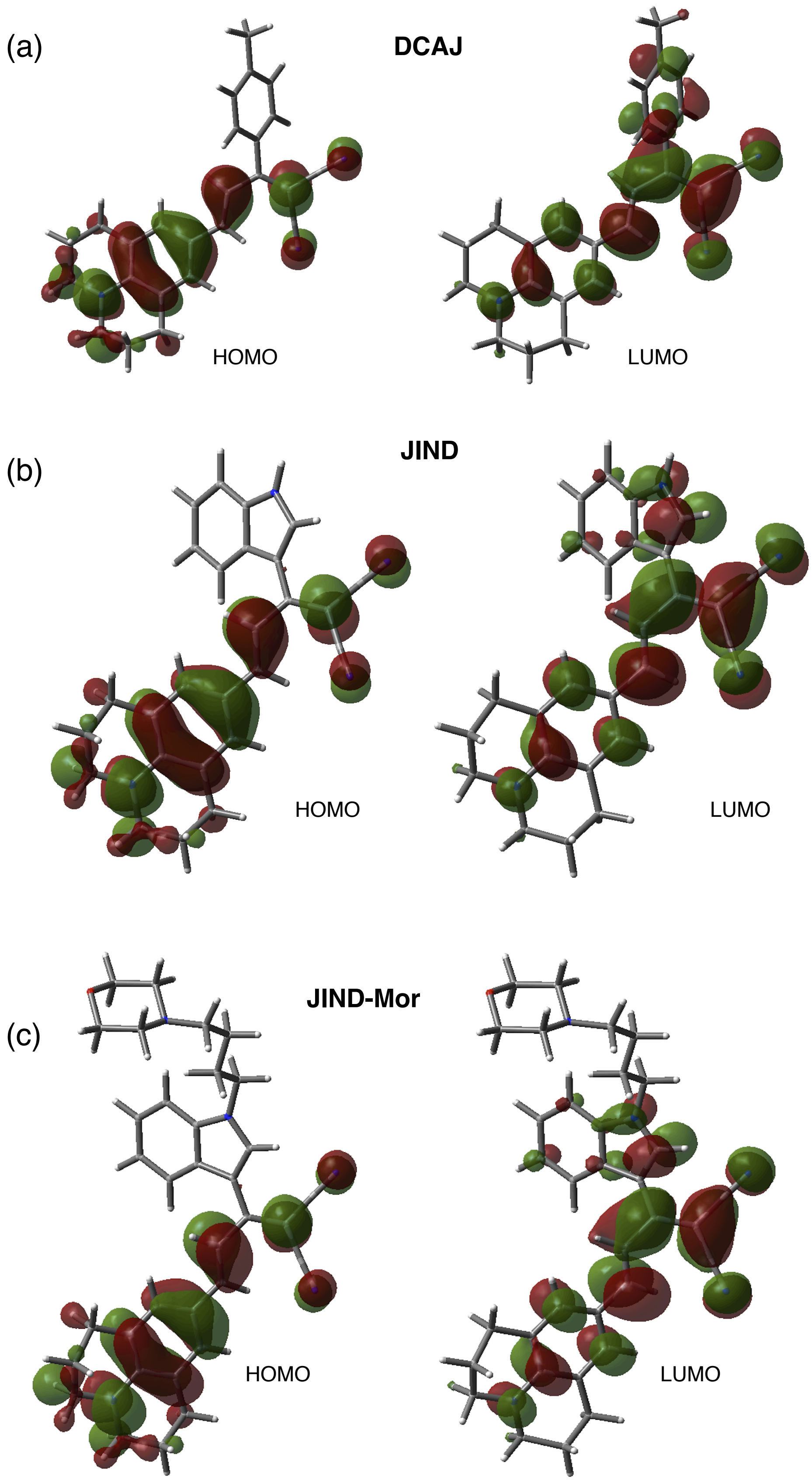

FIGURE S21: FMO pictures of (a) **DCAJ**, (b) **JIND**, and (c) **JIND-Mor**. The FMO pictures clearly indicated the ICT property of these molecular rotors.

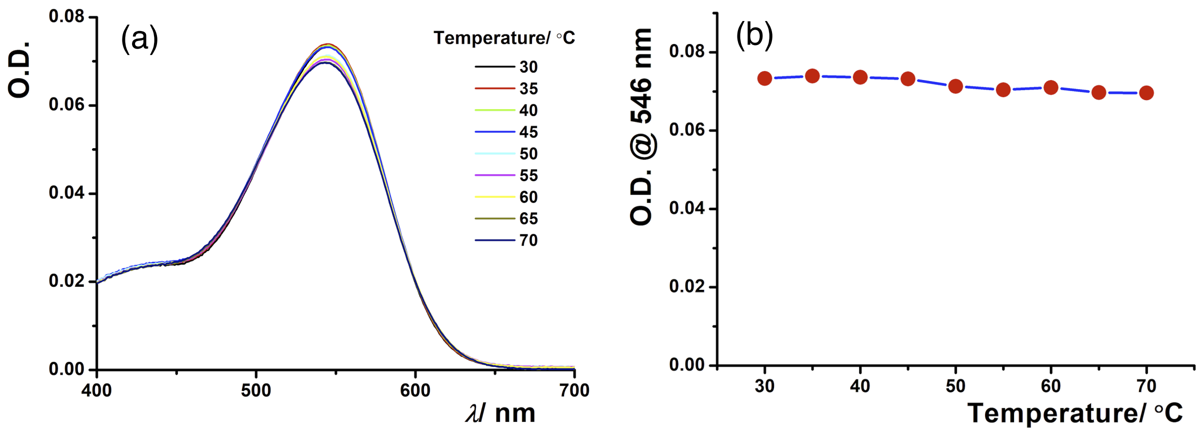

FIGURE S22: (a) Temperature-dependent absorption spectra of **JIND** in EG, (b) absorption maxima Vs temperature plot of **JIND** in EG.

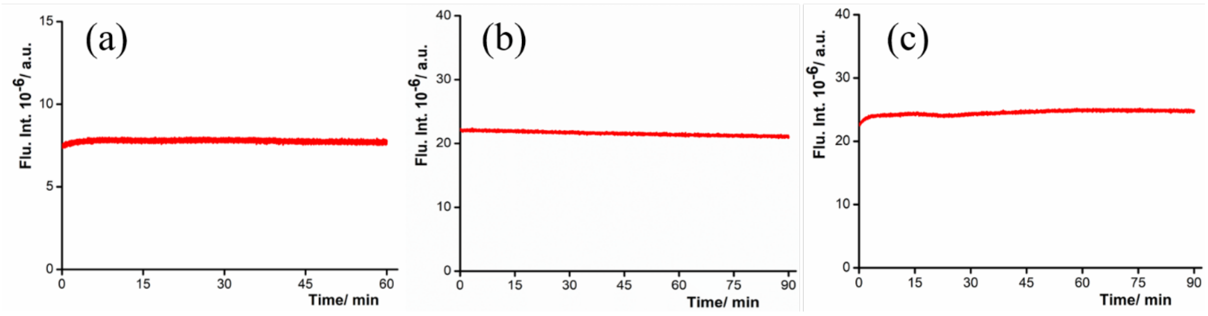

FIGURE S23: The fluorescence intensity at the emission maxima was monitored over time under 540 nm excitation using a 450 W xenon lamp (350 lux) equipped with the fluorolog instrument to assess the photostability of (a) **DCAJ**, (b) **JIND**, and (c) **JIND-Mor**)

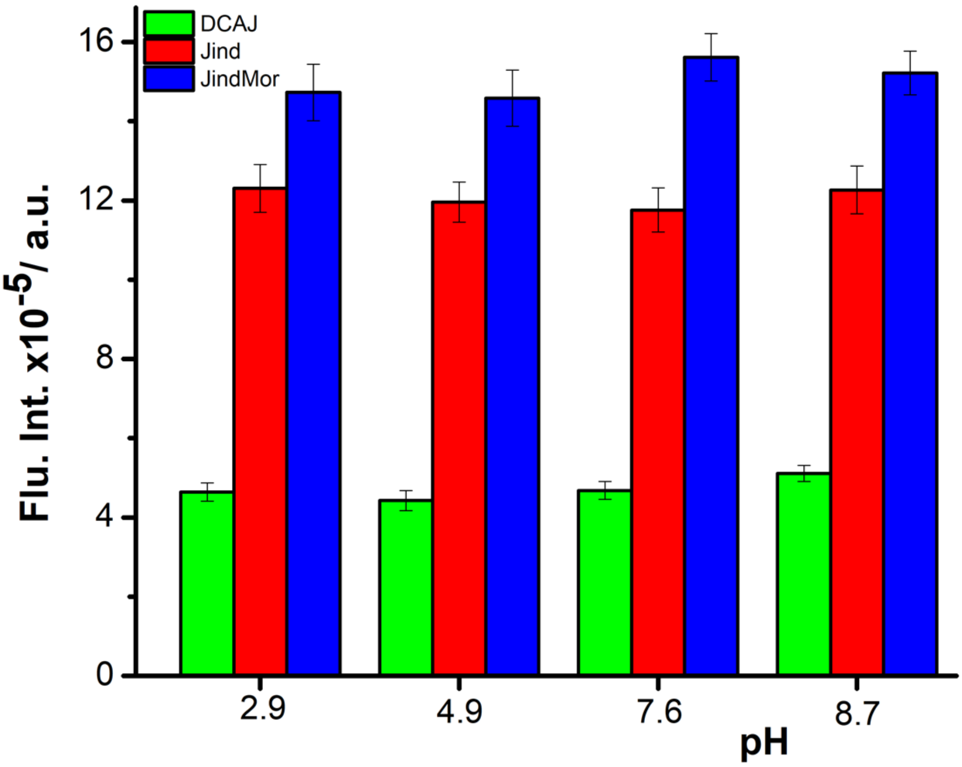

FIGURE S24: The pH stability of the synthesized molecular rotors, there was 5% variation in the measured intensity value.

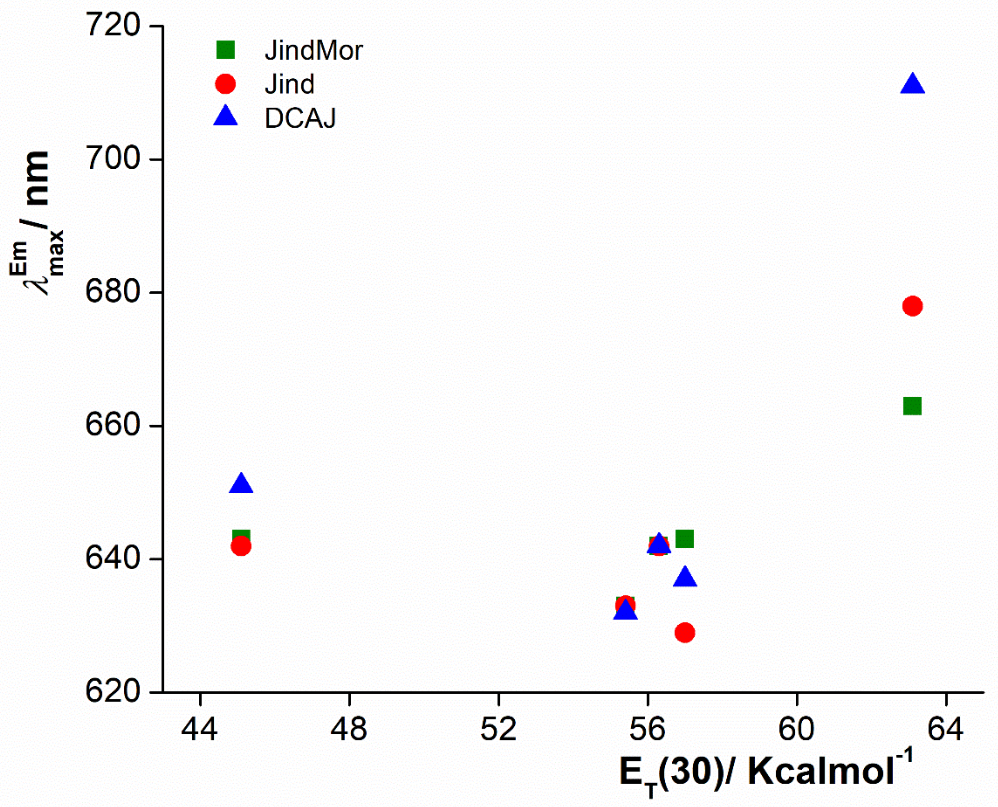

FIGURE S25: A plot of emission maxima with the solvent polarity parameter $E_T(30)$. No linear correlation is observed indicating that there is no general solvent effect.

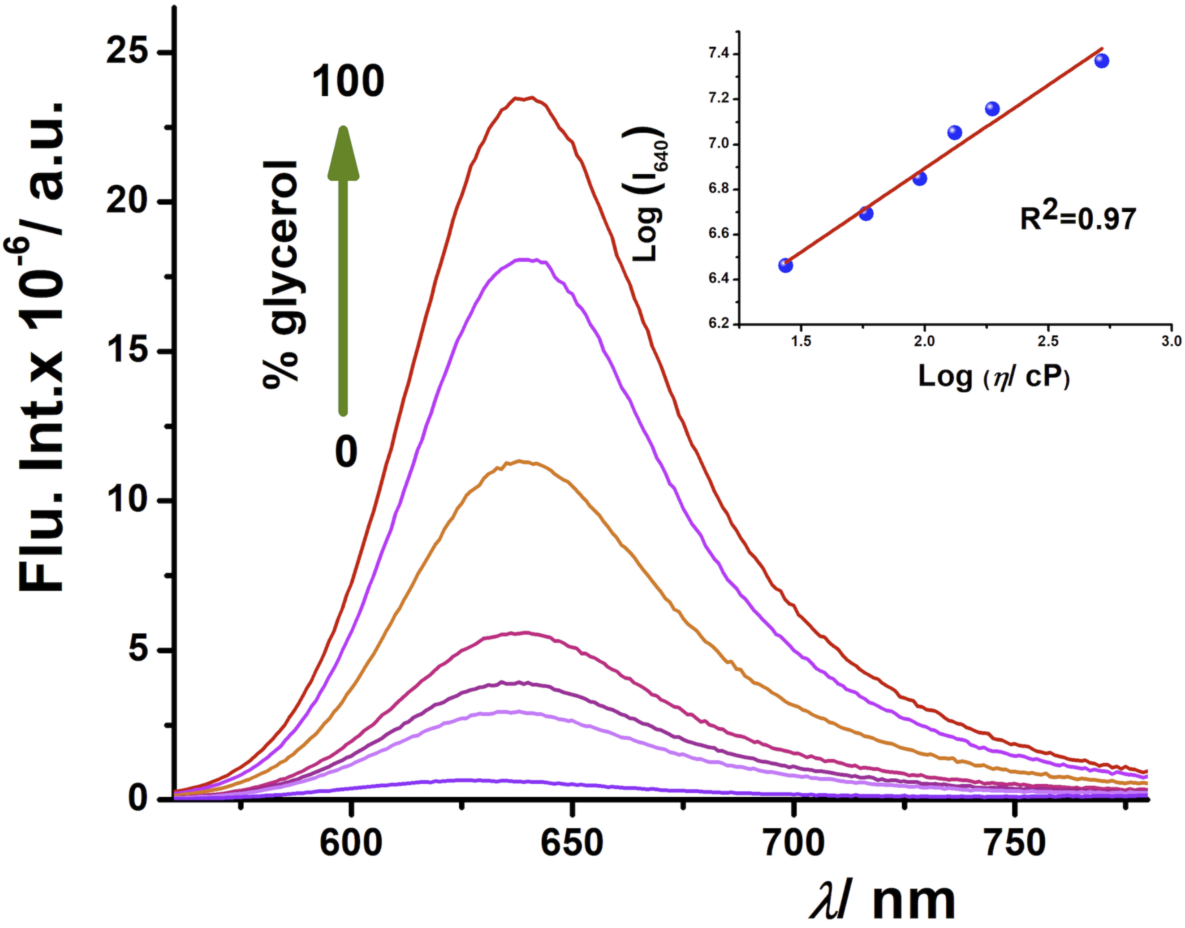

FIGURE S26: Fluorogenic response of 5 μM **JIND** with increasing concentration of glycerol percentage in methanol; inset shows the linear fit of a double logarithmic plot of fluorescence intensity of solution and its viscosity (in centipoise)

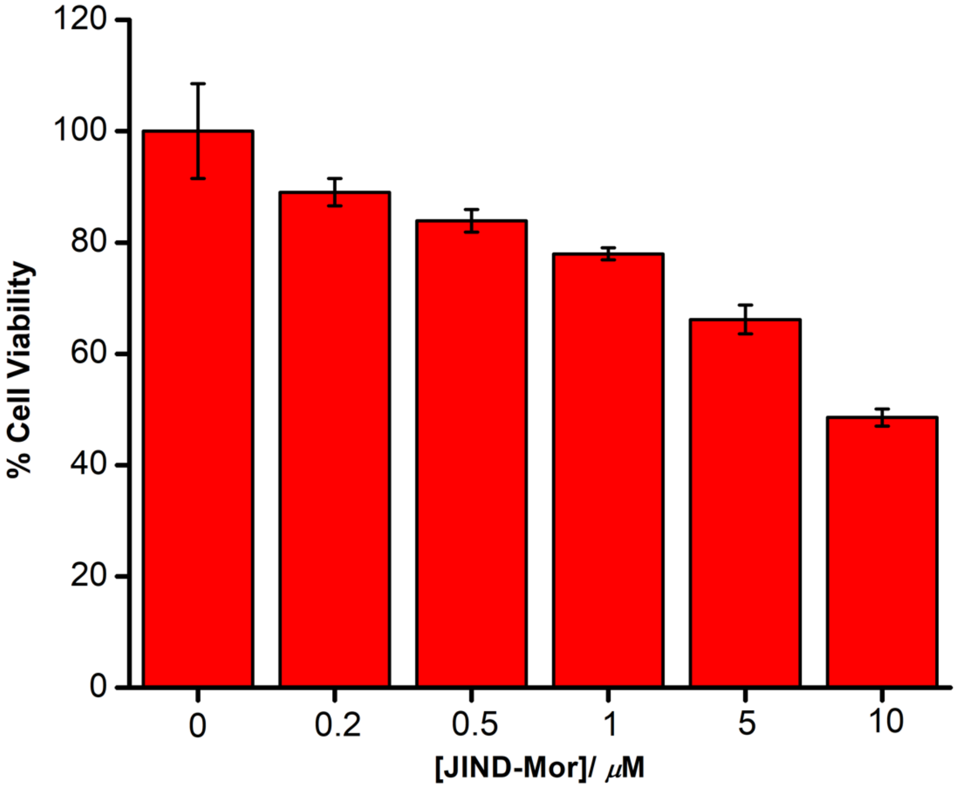

FIGURE S27: MTT assay of **JIND-MOR** incubated for 24 h in BHK-21 cells.

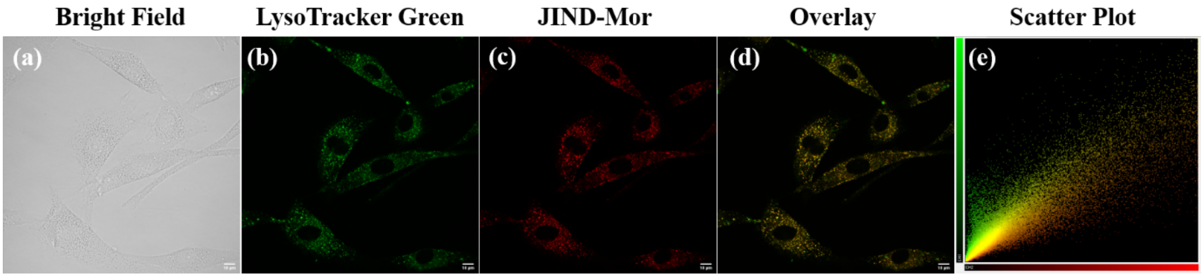

FIGURE S28: CLSM images of U-87 MG cells co-stained with 0.2 μM **JIND-Mor** and 0.3 LysoTracker for 15 min. (a) Bright field, (b) Green Channel (Ex: 488 nm, Em: 500-530), (c) Red Channel (Ex: 561 nm, Em: 570-670), (d) Merge image, and (e) Scatter plot showing Pearson's correlation coefficient of 0.92 ± 0.02. (Scale Bar = 10 μm)

Table S2: Reported molecular viscometers for determination of lysosomal viscosity

Molecular Rotor	$\lambda_{max}^{abs.}$/ nm	$\lambda_{max}^{em.}$/ nm	Stokes shift/ nm	Detection<br><br>Application method	pH tolerance	Reference
**Lyso-V**	488	517	29	Live-cells Flu. Lifetime	No	*J. Am. Chem. Soc.* **2013**, *135*, 2903-2906.
**BDP-1**	488	557	69	Live-cells Flu. Intensity	Yes	*Chem. Eur. J.* **2015**, 21, 3219–3223.
**PIP-TPE**	360	420	60	Live-cells Flu. Intensity	No	*Chem. Sci.* **2017**, *8*, 7593-7603.
**Lyso-B**	550	586	36	Live-cells Flu. Intensity and Lifetime	Yes	*Anal. Chem.* **2018**, *90*, 5873-5878.
**Lyso-AIE2**	405	570	165#	Live-cells Flu. Intensity	Yes	*Anal. Chem.* **2018**, *90*, 8736-8741
**Lyso-NA**	550	610	60	Live-cells Flu. Intensity	Yes	*J. Mater. Chem. B* **2018**, *6*, 580-585
**Lyso-V**	430	515	85	Live-cells Flu. Intensity	Yes	*J. Mater. Chem. B*, ***2018***, 6, *6592-6598*.
**P7-B**	352	419	94	Live-cells Flu. Intensity	Yes	*Dyes and Pigm.* ***2019,*** 188, *108974. Dyes and Pigm.* ***2021,*** 186, *108974*
**Lys-VBOD**	602	637	35	Live-cells Flu. Intensity	No	*Sens. Actuator B-Chem.* **2020**, *304*, 127271.

**Compound 1a**	605	690	85	Live-cells Flu. Intensity	No	*Anal. Chem.* **2020,** 92, 3517-3521.
**Compound 1**	617	649	32	Live-cells Flu. Intensity	Yes	*Sens. Actuator B-Chem.* **2021***, 326,* 128954.
**JIND-Mor**	528	663	135	Live-cells and Living organism Flu. Intensity	Yes	**This work**

Large Stokes-shift is due to aggregation induced emission (AIE) effect.
